# Splice-mediated Variants of Proteins (SpliVaP) – data and characterization of changes in signatures among protein isoforms due to alternative splicing

**DOI:** 10.1186/1471-2164-9-453

**Published:** 2008-10-02

**Authors:** Matteo Floris, Massimiliano Orsini, Thangavel Alphonse Thanaraj

**Affiliations:** 1 CRS4-Bioinformatica, Parco Scientifico e Technologico, POLARIS, Edificio 3, 09010 PULA (CA), Sardinia, Italy

## Abstract

**Background:**

It is often the case that mammalian genes are alternatively spliced; the resulting alternate transcripts often encode protein isoforms that differ in amino acid sequences. Changes among the protein isoforms can alter the cellular properties of proteins. The effect can range from a subtle modulation to a complete loss of function.

**Results:**

(i) We examined human splice-mediated protein isoforms (as extracted from a manually curated data set, and from a computationally predicted data set) for differences in the annotation for protein signatures (Pfam domains and PRINTS fingerprints) and we characterized the differences & their effects on protein functionalities. An important question addressed relates to the extent of protein isoforms that may lack any known function in the cell. (ii) We present a database that reports differences in protein signatures among human splice-mediated protein isoform sequences.

**Conclusion:**

(i) Characterization: The work points to distinct sets of alternatively spliced genes with varying degrees of annotation for the splice-mediated protein isoforms. Protein molecular functions seen to be often affected are those that relate to: binding, catalytic, transcription regulation, structural molecule, transporter, motor, and antioxidant; and the processes that are often affected are nucleic acid binding, signal transduction, and protein-protein interactions. Signatures are often included/excluded and truncated in length among protein isoforms; truncation is seen as the predominant type of change. Analysis points to the following novel aspects: (a) Analysis using data from the manually curated Vega indicates that one in 8.9 genes can lead to a protein isoform of no "known" function; and one in 18 expressed protein isoforms can be such an "orphan" isoform; the corresponding numbers as seen with computationally predicted ASD data set are: one in 4.9 genes and one in 9.8 isoforms. (b) When swapping of signatures occurs, it is often between those of same functional classifications. (c) Pfam domains can occur in varying lengths, and PRINTS fingerprints can occur with varying number of constituent motifs among isoforms – since such a variation is seen in large number of genes, it could be a general mechanism to modulate protein function. (ii) Data: The reported resource (at ) provides the community ability to access data on splice-mediated protein isoforms (with value-added annotation such as association with diseases) through changes in protein signatures.

## Background

Human genome encodes a surprisingly low number of genes; however a large transcriptome has been reported for human [1-3]. Alternative splicing of exons, during the processing of pre-mRNA, is a major contributor to the diversity seen in transcriptome and proteome [4,5]. Transcript isoforms from a gene often encode functionally diverse protein isoforms [5-9]. It has been reported that gene regulation through alternative splicing is more versatile than that through promoter activity [1,10]. The many other mechanisms that the cell uses to introduce variation at gene or transcript or protein level (such as RNA editing and post-translational modifications) are themselves affected by alternative splicing (for example, introduction of protein domains that bring about post-translational modifications [5]).

Alternative splicing leads to variants of proteins with diverse changes that can range from profound effects to fine modulation of protein activity [11]. An example that illustrates drastic change can be seen among the isoforms of caspase-9 protease: the constitutive form of the protein induces apoptosis, while its shorter isoform acts as an inhibitor [12]. An example that illustrates fine modulation can be seen among the isoforms of AT1: the protein product of human AT1 (angiotensin II type 1 receptor) gene binds to angiogenesis II (Ang II) hormone peptide; four transcript isoforms have been identified for hAT1 gene that essentially leads to two protein isoforms differing from one another by a 32-amino acid extension at the N-terminal; the shorter isoform has higher affinity to the hormone peptide than the longer isoform; the potency of the Ang II response varies depending on the relative abundance of these two protein isoforms [13].

Splice-mediated changes at transcript level can be seen in both the untranslated and the coding regions. Changes in the untranslated regions can lead to inclusion/exclusion/modification of RNA regulatory elements responsible for the translatability of the mRNA. Changes in coding regions can lead to insertion/deletion/substitution of amino acid residues in the encoded proteins and thereby bring about differences in the constituent functional/structural motifs; such changes in a protein can alter its binding properties (*e*.*g*. in terms of the binding affinities and the types of binding molecules), can influence its intracellular localization (*e*.*g*. in terms of effecting changes on signal peptides or localization signals), can modify its enzymatic activity (*e*.*g*. in terms of effecting changes in substrate specificity, catalytic properties or affinity), and can modify its intrinsic stability (*e*.*g*. by introducing regions for autophosphorylation or signals for cleavage) [5,14]. The effects due to such changes can range from a complete loss of function to very subtle activity modulation. The 3-dimensional structure of a protein can be drastically altered by splice-mediated deletion of large regions or even of small regions that are part of long-range structural stabilizations; modeling studies [1,6] have reported that up to 67% of alternative spliced isoforms can show significant alterations in regions that form the core of protein structure and thereby large conformational differences. Tress *et al *[15] find little evidence as to whether a majority of protein isoforms have a role as functional proteins.

Missplicing events can cause or contribute to human diseases. At least 15% of human disease-causing mutations occur at splice sites [16]; mutations and genetic variations can alter the splice site signals and splice regulatory elements to mediate formation of alternate transcripts and protein isoforms [17-20]. Aberrantly spliced isoforms play a direct role in transformation, motility and metastasis of tumor tissue; array and RT-PCR experiments [21] confirm that differentially expressed transcripts correlate extremely well with known cancer genes and pathways; and cancer-specific novel splice isoforms have been identified in human expressed sequence collections [22]. It is important to characterize functional changes in protein isoforms and to understand the association between the pathological states of the cell and the synthesized protein isoforms; this will help in developing novel peptide-based probes and targets for identifying and treating human diseases.

We considered two large data sets of splice-mediated protein isoform sequences from human and delineated differences in signatures among the isoforms – the data sets of examined protein isoforms are of two different types, namely one from a database of manually curated isoforms and the other from computationally predicted splice isoforms as seen in EST resources. Changes among protein isoform sequences are discussed in terms of inclusion/exclusion/alternation/truncation of protein signatures (domains as defined by Pfam [23] and fingerprints (as defined by PRINTS [24]) as well as in terms of lack of annotation for signatures. We present to the community the resultant database (SpliVaP) containing information on changes in the composition and structure of signatures among protein isoform sequences (with value-added annotations such as associations with diseases).

## Methods

### Data on protein isoform sequences

For data on protein isoform sequences, we considered two independent sources – one based on manually curated database of splice isoforms, and another based on computational delineation of splice isoforms from EST sequences.

#### Manually curated data set

For curated data on splice-mediated protein isoforms, we used Vega (The vertebrate genome annotation) database [25] as available from . Vega acts as the central repository for the majority of genome sequencing centres to deposit their annotation of human chromosomes. The manual curation of the human genome in Vega is thus performed by an international group of collaborators (see  for details). We used release v31 (Apr 2008) of the Vega database for homo sapiens for the current study. The data set was cleaned for redundant protein isoform sequences – if two or more protein isoform sequences from a gene are identical to one another, only one was retained. The such cleaned data set comprises 33502 protein isoforms from 9649 human genes.

#### Computationally predicted data set

We extracted data on splice-mediated protein isoforms from Alternative Splicing Database (ASD) [26] as available from . Release 3 [27] of the ASD database for *homo sapiens *was used for the current study; the data set was cleaned for redundant protein isoform sequences – if two or more protein isoform sequences from a gene are identical to one another, only one was retained. The such cleaned data set comprises 27,241 protein isoforms from 7,664 human genes. A brief note on the derivation of data on protein isoform sequences by the ASD pipeline is in order here. ASD pipeline uses EST/mRNA transcript sequence data to firstly identify isoform splice patterns of a gene; nucleotide sequence of an isoform splice pattern is derived by extracting the appropriate exon regions from the gene sequence; the relevant protein sequence corresponding to such a splice pattern is then derived from the nucleotide sequence of the splice pattern by adopting one of the following two approaches: (a) mRNA evidence: When one of the transcript sequences confirming the splice pattern is an mRNA with annotation for coding information (*i*.*e*. start and end of translated region), the information is used to translate the splice pattern sequence onto protein sequence; such a derived protein sequence is annotated as having mRNA experimental evidence; it is often the case that such annotated mRNA entries are associated with protein sequence entries in UniProt [28] database. (b) ASD prediction: This is for those splice patterns that are confirmed entirely by EST sequences or by mRNA with no annotation for coding information. All regions starting with ATG codon from the splice pattern sequence are assessed for translatability; length of the translated peptide and the overall match to a reference protein are assessed. Thresholds based on ATG-context scores [29] (as detected using a set of experimentally determined translation initiation codons on human mRNAs) are applied. Longest open reading frame is then selected to give rise to translated protein sequence.

### Annotation of protein isoform sequences for PRINTS fingerprints and Pfam domains

#### Annotation for PRINTS fingerprints

A PRINTS fingerprint [24] is a group of conserved motifs used to characterize a protein family. The fingerprint concept is based on the fact that sequences of proteins from a family hold in common subsequences (sequence motifs) that usually relate to key functional elements or core structural elements; the motif is any conserved element seen in the alignment of sequences forming a family. InterProScan [30] is a tool that identifies fingerprints in a given protein sequence. Annotation by InterProScan for a fingerprint does not necessarily mean that all the constituent motifs of the fingerprint are seen in a given protein sequence. We aligned the protein isoform sequences from our data sets with PRINTS fingerprint signatures using InterProScan. We retained only those alignments with an E-value ≤ 10^-5^. Annotation for fingerprints can produce partial or total overlap in fingerprint definitions along the length of the sequences; such isoforms numbered 2257 in the case of Vega and 711 in the case of ASD.

#### Annotation for Pfam domains

Pfam is a large collection of multiple sequence alignments and hidden Markov models covering many common protein domains and families. Alignments of the protein isoforms with Pfam definitions were performed by using HmmPfam [31,32]. We retained only those annotations with an E-value ≤ 10^-5^. Annotation of protein sequences for Pfam domains can produce partial or total overlap in domain definitions along the length of the sequences; such isoforms numbered only 173 in Vega data set, and 405 in ASD data set.

### Examining the protein isoforms for changes in signatures (fingerprints or domains)

**Figure 1:**
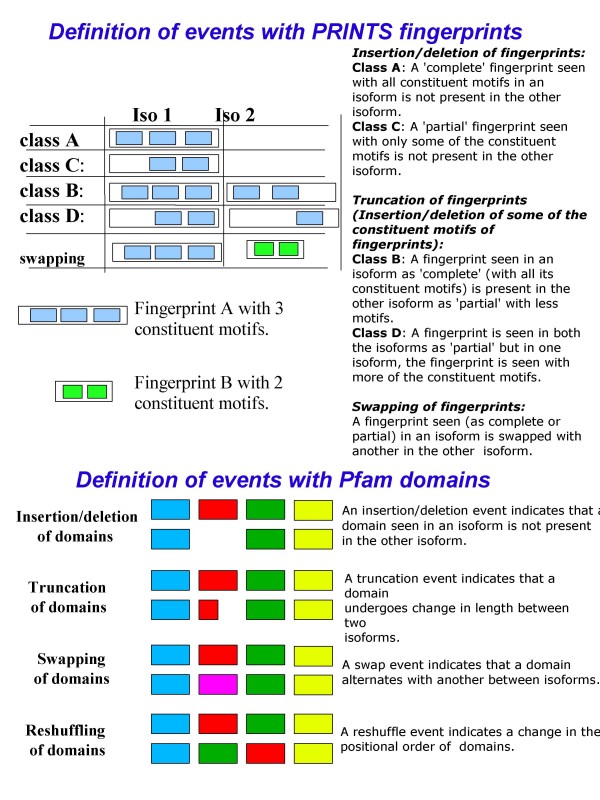
Definitions of splice-mediated changes in the annotation for PRINTS fingerprints and Pfam domains among protein isoforms.

For every gene, we firstly identified a *reference protein *which is the longest of the expressed protein isoforms; choosing the longest protein as reference is justified by an observation that in only < 5% instances of genes, the longest peptide had fewer Pfam domains or PRINTS signatures than the other isoforms. We then identified changes in signatures as seen between such a reference protein and each of the protein isoforms. Definitions of such splice-mediated changes are illustrated in Figure 1. Splice-mediated changes in an isoform is identified by firstly performing a dynamic alignment of the signature pattern of the isoform with that of the reference protein. Three types of alignments can result – (i) Same Patterns: the composition and order of the signatures are same in both the reference and isoform protein; however, this set of isoforms can still contain truncation events (change in length of a Pfam domain or change in the number of constituent motifs of a fingerprint at an aligned position). (ii) Totally Different Patterns: none of the signatures seen in the reference protein is present in the isoform; and (iii) Patterns with Changes: there are changes in the composition and order of signatures between the reference and isoform protein; however, at least one common signature could be seen. The cases of Totally Different Patterns were not taken up for further analysis because they can be results of the artifacts in peptide delineations or results of strict criteria used to annotate for signatures. The other two types are taken up for further characterization as below: (i) The Patterns with Changes are examined further for specific types of changes (such as insertion/deletion, truncation, swap, and reshuffle) by scrutinizing the aligned positions; and (ii) the Same Patterns are examined further for truncation event. In our alignment schema, a position is occupied either by a signature or by a gap; the signature is characterized by the name, number of constituent motifs (in the case of fingerprints) or by the length in amino acids residues (in the case of domains).

#### Insertion/Deletion and Truncation of PRINTS fingerprints

A PRINTS fingerprint is defined by a collection of constituent motifs. A variety of changes in fingerprint patterns can be seen among protein isoforms – none or only few or all of the constituent motifs of a fingerprint predicted in an isoform can be seen in the other isoforms. We categorized insertion/deletion changes seen between two isoform sequences onto 4 classes as defined below: Class A event: A 'complete' fingerprint seen (with all its constituent motifs) in an isoform is totally lost in the other isoform; Class B event: A 'complete' fingerprint (with all its constituent motifs) is seen in an isoform while some of its constituent motifs are lost in the other isoform; Class C event: A 'partial' fingerprint seen (with only some of the constituent motifs) in an isoform is not seen in the other isoform; Class D event: Both the isoforms possess the fingerprint as 'partial', but one isoform shows more of the constituent motifs. We term the Class A and C events as Insertion/Deletion of fingerprints, and Class B and D events as Truncation of fingerprints.

#### Insertion/deletion of Pfam domains

A gap in the aligned position leads to identification of domain insertion/deletion. We observe in our data sets that a considerable number of protein isoforms are annotated with successive repeats of a domain. Such repeats can be collectively considered as one entity of domain; in instances of insertion/deletions of some of the repeats but not all, we annotate the change as Insertion/Deletion – Reduction of repeats; and when all the repeats are involved, we annotate the change as Insertion/Deletion – All repeats. We find that delineation of events is ambiguous when a protein isoform is annotated with repeats of domains, and we tend to ignore such instances for identifying events.

#### Truncation of Pfam domains

Pfam domains are derived from alignments of a representative set of sequences. For each domain are available manually verified multiple alignments, hidden Markov Models (HMM) and full-alignments. A single protein can belong to several Pfam families. For each database search, sequences that score more than the family-specific threshold are aligned to the HMM profile automatically to make a full alignment. Thus domains can have more than one defined region that can differ in length across taxonomy; it is often the case that a domain can have a large defined region of sequence on eukaryotic proteins as compared to their homologs in prokaryotes. We examined the lengths of every domain from aligned positions; and the domain is considered to undergo truncation when the lengths differ by more than 5 amino acid residues at an aligned position.

#### Swapping of signatures among protein isoforms

A swap event is indicated by two gaps at successive aligned positions (one from each of the aligned reference and isoform protein sequence). A note on swap events with *PRINTS fingerprints: *A fingerprint seen (either as 'complete' or 'partial') in reference protein is swapped with another fingerprint ('complete' or 'partial') in the isoform sequence.

#### Reshuffling of signatures among protein isoforms

A reshuffle event is identified when the order of occurrence of 2 or more signatures as seen in the reference protein is reversed in the isoform sequence.

#### Quality check on the detection of events

The alignments of the signature patterns were manually curated. Detected events from the alignments were double-checked for correctness by developing scripts that implement heuristics-based methods.

### Associations of isoforms with structural data

In order to provide to community structural data corresponding to protein isoforms, we performed BLAST [33] alignments of the protein isoform sequences with the sequences of structural entries in the Macromolecular Structure Database (MSD) [34]. *S*tructural data for a protein isoform sequence from our data set is considered to be present in MSD, if the coverage ≥ 98.0% (*i*.*e*. at least 98% of the residues from the query sequence aligns with the target sequence in MSD with no gaps) and the identity is ≥ 98.0% (*i*.*e*. at least 98% of aligned positions are occupied by same amino acid residue in both the query and target sequences). For such isoform sequences, we made associations with MSD entries in our database.

### Association with genetic disorders

Information on gene associations with diseases was obtained from the resource of Online Mendelian Inheritance in Man (OMIM) [35]. For each of the genes thus associated, we extracted the PubMed Identifiers of the journal articles cited in the OMIM entry. We then extracted all the Mesh terms associated with these PubMed Identifiers. These mesh terms and the OMIM terms were attributed as keywords describing the association of genes to diseases.

### Examination of transcript isoforms (encoding the protein isoforms) for susceptibility to nonsense-mediated decay (NMD)

This was done for splice isoforms from the ASD data set. Splice patterns corresponding to the protein isoforms were extracted from the ASD database. If the position of stop codon is seen mapped more than 50 nucleotides upstream of the last exon-exon junction of the splice pattern, then such a splice pattern is considered as a possible target for nonsense-mediated decay [36-38].

## Discussion

### Varying degrees of annotation of protein isoforms for Pfam/PRINTS signatures

**Figure 2:**
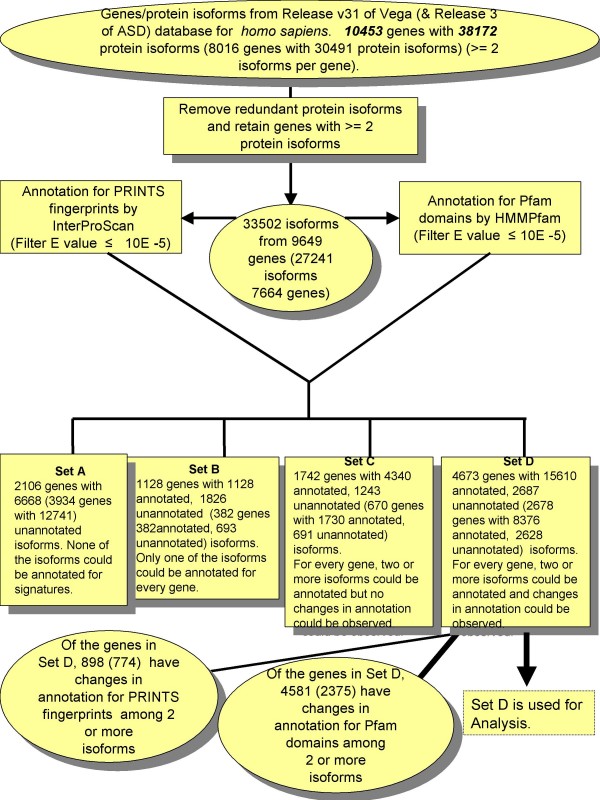
**Flow of data (on genes and protein isoforms) through methodological steps adopted to derive the Set D used for characterizations.** The numbers given in red correspond to the ASD data set, and those given in print colour correspond to the Vega data set. The number of genes in Set D forms 44.7% (33.4% in the case of ASD) of the genes from the start-up data set, the number of (PRINTS and Pfam) annotated protein isoforms and unannotated protein isoforms form 41% and 7% (27.5% and 8.6% in the case of ASD), respectively of the isoforms from the start-up data set.

We considered two data sets (one from Vega and the other from ASD) of human genes with at least two or more protein isoform sequences identified for each gene; the protein isoform sequences were then examined for the presence of Pfam/PRINTS signatures. This exercise resulted in four distinct data sets (See Figure 2 for flow of data across different steps leading to the following distinct data sets):

Set A (with Vega: 2106 genes; 0 annotated isoforms; 6668 unannotated isoforms from all the 2106 genes; with ASD: 3934 genes; 0 annotated isoforms; 12741 unannotated isoforms from all the 3934 genes): This set contains those genes for which none of the reported protein isoforms could be annotated for Pfam/PRINTS signatures. The reasons for lack of annotation may include (i) that the criteria on thresholds used in the methodologies to review the alignments of Pfam and fingerprints with the protein isoform sequences is strict; and (ii) that examining the sequences only for Pfam domains and PRINTS fingerprints is not enough and further resources may need to be used.

Set B (with Vega: 1128 genes; 1128 annotated isoforms from all the 1128 genes; 1826 unannotated isoforms from all the 1128 genes; with ASD: 382 genes; 382 annotated isoforms from all the 382 genes; 693 unannotated isoforms from all the 382 genes): This set contains those genes for which only one of the protein isoform sequences could be annotated and the other isoforms lack annotation. It is possible to say that the only annotated isoform represents the constitutively expressed protein product and that any of its variants lack functions (within the constraints highlighted above for Set A).

Set C (with Vega: 1742 genes; 4340 annotated isoforms from all the 1742 genes; 1243 unannotated isoforms from 590 genes; with ASD: 670 genes; 1730 annotated isoforms from all the 670 genes; 691 unannotated isoforms from 359 genes): This set contains those genes for which two or more protein isoform sequences could be annotated but no decipherable changes could be observed in the annotation for signatures between the reference protein and any of the isoforms. Though the annotated isoforms are different from one another in amino acid sequence, they do not exhibit any change in signatures – the possible reasons are that (i) the amino acid differences are small and do not affect the domain/fingerprint definitions; and (ii) the regions that are different among the isoforms are not annotated for domains/fingerprints and hence no change in signatures is seen among the isoforms.

Set D (with Vega: 4673 genes; 15610 annotated isoforms from all the 4673 genes; 2687 unannotated isoforms from 1385 genes; with ASD: 2678 genes; 8376 annotated isoforms from all the 2678 genes; 2628 unannotated isoforms from 1346 genes): This set contains those genes for which two or more protein isoform sequences could be annotated and changes in signatures could be seen between the annotated reference and at least one of the isoforms. Some of the isoforms of a subset of genes lack annotation.

The observed varying degree of annotation indicate lack of signatures in all or some of the protein products from certain genes; such a lack of annotation has been observed by other researchers as well – *e*.*g*. based on the work using full-length human cDNAs from H-invitational transcriptome data, Takeda *et al *[39] find that in 20% instances of alternatively spliced human genes, the protein products lacked annotation for protein motifs. For the work undertaken in this study (splice-mediated changes in protein isoforms), Set D is the appropriate resource as it presents a list of genes in which two or more protein isoforms could be annotated for Pfam/PRINTS signatures and changes in signatures could be deciphered among the protein isoforms. In all the subsequent discussions, the Set D is used.

### Splice-mediated events with PRINTS fingerprints among protein isoforms

#### Overlapping annotation for fingerprints and the effects that alternative splicing has

We found 610 peptides in Set D of the Vega data set to be annotated in an overlapping manner, 513 of which have the overlapping fingerprints from same top-level classification (in ASD data set, the numbers are 552 and 472). We raised a question as to how often alternative splicing removes overlaps? We examined isoform pairs where one or both the partners are from this set of peptides with overlapping annotation. In 1548 instances of 2036 such pairs from Vega data set, alternative splicing removed the overlapping fingerprint(s) (in ASD data set, the numbers are 788 of 1242) – this phenomenon can be considered as an event by itself (though it can be treated as fingerprint insertion/deletion).

#### Insertion/deletion, and truncation of fingerprints

Changes in fingerprints among the annotated protein isoforms were seen in a data set of 898 Vega and 774 ASD genes. Classification of PRINTS events as insertions/deletion of fingerprints (Classes A and C events), and truncation (Classes B & D events) (see the section on Methods) is informative in terms of severity of the effects on the function. Insertion/deletion events, where a fingerprint (seen with all or some of the constituent motifs) is totally lost between two isoform sequences, may bring severe effects as compared to the other type (namely truncation, where the fingerprint can still be seen in both the isoform sequences *albeit *with differing number of constituent motifs). Our data sets show that truncation of fingerprints occurs in more number of genes than insertion/deletion of fingerprints; truncation occurs in 848 Vega (in 734 ASD) genes while insertion/deletion occurs in 242 Vega (in 226 ASD) genes. Since truncation events are seen in a large number of genes, it could be that truncation of fingerprints is a mechanism to modulate protein functionalities. It is to be mentioned here that the presented fingerprint truncation phenomenon is different from the N-terminal and C-terminal protein shortening (or truncation) that the splicing community talk about – it is usually the case that in such protein shortenings, a signature is completely lost. It is significant if the observed fingerprint truncations are often seen not as part of the N- or C-terminal shortenings but are seen in the internal regions of the shorter isoforms. We examined how often the observed truncations of fingerprints are results of N-, or C-terminal protein shortenings as opposed to genuine internal truncations. We define the fingerprint truncation as part of N- or C-terminal protein shortening, if the number of amino acid residues separating the truncated end of the fingerprint from the corresponding terminal of the shorter protein by less than 5 amino acids. The ratios of observed truncations were seen as (part of N-terminal shortening : genuine internal : part of C-terminal shortening = 1 : 7.6 : 1.5 in the case of ASD genes, and 1 : 8.8 : 1.9). Thus the fingerprint truncations are not mainly due to alternative start/stop codons. Table 1 lists the top-level classifications of fingerprints that often undergo insertion/deletion and truncation events in our data set; it is seen that the major classes of fingerprints that undergo insertion/deletion/truncation events are receptors, enzymes (hydrolases, oxidoreductases, and transferases), transport proteins, structural proteins, RNA- or DNA-associated proteins, and 'Domain' signatures (such as those of SH2/SH3, Ankyrin, Apple and Kringle domains – see [40] for a list). The top-ranking fingerprints from the above-mentioned classes are signatures of: SH2 domain signature, C4-type steroid receptor zinc finger signature, Steroid hormone receptor signature, P450 superfamily signature, Neurotransmitter-gated ion channel family signature, Secretin-like GPCR family signature, Tyrosine kinase catalytic domain signature, and Short-chain dehydrogenase/reductase (SDR) superfamily signature (See additional file 1: Additional File 1 for a list of top 10 frequently observed fingerprints that undergo insertion/deletion event among protein isoforms).

**Table 1:** Classifications of fingerprints involved in insertion/deletion and truncation events.

Top-level PRINTS classifications of fingerprints	No. of observed events* affecting			
	the whole fingerprint^&^		some of the constituent motifs of the fingerprint^$^	
	Type A – insertion/ deletion of a 'complete' fingerprint	Type C – insertion/ deletion of a 'partial' fingerprint	Type B – truncation of a 'complete' to 'partial' fingerprint	Type D – fingerprint is partial in both the isoforms and possess differing number of constituent motifs
Receptors	61 (23%)	23 (*8%*)	159 (**62%**)	13 (*5%*)
	64 (22%)	33 (*11%*)	153 (**54%**)	33 (*11%*)
Enzymes: Hydrolases	11 (*9%*)	4 (*3%*)	90 (**79%**)	***8****(7%)*
	13 (*10%*)	2 (*1%*)	97 (**73%**)	19 *(14%)*
Transport proteins:others	51 (27%)	15 (*7%*)	100**(53%)**	22 (*11%*)
	27 (19%)	21 (*15%*)	77**(55%)**	15 (*10%*)
Enzymes: Oxidoreductases	17 (18%)	11 (*11%*)	39 (**41%**)	27 (28%)
	11 (*10%*)	19 (18%)	40 (**38%**)	34 (32%)
Enzymes: Transferases	13 (*14%*)	3 (*3%*)	69 (**78%**)	3 (*3%*)
	17 (18%)	5 (*5%*)	63 (**68%**)	7 (*7%*)
Structural proteins	26 (*15%*)	12 (*7%*)	107 (**62%**)	26 (*15%*)
	13 (*14%*)	6 (*6%*)	49 (**55%**)	20 (22%)
RNA- or DNA-associated proteins	17 (*15%*)	3 (*2%*)	67 (**62%**)	*20* (18%)
	17 (23%)	2 (*2%*)	44 (**60%**)	10 *(13%)*
PRINTS 'Domains' signatures	20**(36%)**	13 (23%)	21 (**38%**)	*1(1%)*
	26**(52%)**	4 (*8%*)	17 (**34%**)	3 *(6%)*
Cytokines and growth factors	4 (*14%*)	2 (*7%*)	17 (**62%**)	4 (*14%*)
	6 (18%)	3 (*9%*)	22 (**66%**)	2 (*6%*)
Protein secretion and chaperones	0 (*0%*)	2 (14%)	8 (**57%**)	4 (28%)
	0 (*0%*)	6 (26%)	11 (**47%**)	6 (26%)

*, Events of same insertion/deletion type involving fingerprint members of a PRINTS classification observed among multiple pairs of isoforms from a gene are counted as one. Per every type of insertion/deletion event, the observed numbers involving the five top scoring fingerprint PRINTS classification are underlined. In brackets are given values on what fraction of events involving the PRINTS classification is of the given insertion/deletion type – values > = 35% are in bold; values < = 15% are in italics. Values for Vega data set are given in line 1 and values for ASD data set are given in line 2 in every row.

^&^, A fingerprint seen as either 'complete' (with all the constituent motifs) or 'partial' (with only some of the constituent motifs) in an isoform is deleted in the other isoform.

^$^, Some of the constituent motifs of a fingerprint ('complete' or 'partial') in an isoform are deleted in the other isoform.

#### Swapping, and Reshuffle of fingerprints

In addition to the above-mentioned insertion/deletion and truncation events, we looked for other events such as swap (where a fingerprint seen, either as 'complete' or 'partial', in a protein sequence is swapped with another in the isoform sequence), and reshuffle (where the positional ordering of fingerprints as seen in a protein sequence is reversed in the isoform sequence). Just one instance of swap was seen (in ASD data set; Vega data set showed 4 instances but they are ambiguous because the protein isoform is annotated with fingerprints that overlap in positions) and one instance of reshuffle (in Vega data set – reshuffling among SH2DOMAIN and SH3DOMAIN) event was observed.

### Splice-mediated events with Pfam domains among protein isoforms

#### Relative frequencies of different splice events with domains

We observed splicing events associated with Pfam domains in 4581 Vega and 2375 ASD genes. Truncation in domain length is the most predominant event (at 54% of the instances of Vega protein isoform pairs, at 35% of the instances of ASD protein isoforms pairs) followed by insertion/deletion of domains (at 46% of the instances of Vega protein isoform pairs, at 29% of the instances of ASD protein isoform pairs). Swapping of domains occurred in few instances (56 Vega isoform pairs and 9 ASD isoform pairs). Reshuffling of domains was observed in just one pair of protein isoforms. Occurrence of truncation events in a large number of instances can probably be associated with regulation, while insertion/deletion events can be associated with a regulation activity ranging from fine-tuning to drastic changes (depending on the nature of the domain and the context of the splicing event).

#### Truncation of domains

Examination of protein isoform sequences for domains that are expressed in different lengths revealed that the data on domain truncations is more complex than we expected; even when a region (corresponding to a domain) is shortened by large extents, it is annotated by HmmPfam for the same domain. Table 2 lists Pfam domains that frequently undergo truncation (show different lengths in protein isoforms) as ranked by the number of genes encoding the domain in different lengths among the protein isoforms. In each of the cases of listed domains, a high percent fraction of the genes encoding the domain exhibit domain truncation. In each case of domains, a large number of variations in lengths is observed; highest number of variations is seen in the cases of Pkinase (52 variants), MFS_1 (33 variants), Serpin (30 variants), Trypin (23 variants) and Filament (23 variants) domains. Examination of data on the extent of variation in the lengths of regions, that could still be annotated for same domains, reveals that the variation can be extensive – e.g. a variation of more than 100 amino acid residues could be seen in the cases of domains Pkinase, MHC_I, Filament, PH, etc. Since a large number of domains (Vega: 1552 of 2057 distinct annotated domains; ASD: 1149 of 1592 distinct annotated domains) are seen to undergo truncations in a large number of genes (Vega: 3532 of 4581 genes; ASD: 1779 of 2375 genes), it could mean that truncation could be a mechanism to modulate the processes in which they are involved. As in the case of fingerprint truncations, we observe here that domain truncations are not mainly due to N- or C-terminal shortenings of the proteins; the ratios of observed domain truncations are seen as (part of N-terminal shortening : genuine internal : part of C-terminal shortening = 1.5 : 4 : 1). Thus the domain truncations are not mainly due to alternative start/stop codons.

**Table 2:** Pfam domains that are frequently truncated among protein isoforms.

Domain	No. of genes that encode the domain in different lengths among the protein isoforms (& as percentage fraction of genes encoding the domain in the protein isoforms)^$^	Count of unique domain lengths ^@^	Variation in lengths of the domain among isoforms	
			Minimal length	Maximal length
Pkinase	116 (77%)	52	23	572
	3 (8%)	5	199	486
C1-set	47 (43%)			
	Not seen in ASD	8	30	89
Ras	38 (92%)			
	Not seen in ASD	***21***	25	192
MHC_I	35 (70%)	7	36	178
	3 (60%)	5	91	178
Trypsin	29 (60%)	***23***	32	***261***
	20 (66%)	***14***	104	***261***
ABC_tran	29 (80%)	15	55	***197***
	11 (68%)	9	80	199
Filament	26 (92%)	23	34	452
	4 (100%)	6	142	400
PH	24 (25%)	15	26	241
	6 (15%)	5	84	134
MFS_1	23 (92%)	33	82	537
	6 (54%)	7	326	426
Serpin	22 (100%)	30	31	424
	10 (100%)	13	140	378
P450	22 (100%)	22	86	463
	13 (72%)	20	187	486
Proteasome	22 (100%)	11	29	191
	9 (100%)	8	117	187
7tm_1	22 (70%)	24	25	459
	16 (48)	17	149	388
Ion_trans	21 (63)	18	27	280
	1 (10%)	2	208	220
RRM_1	20 (46%)	9	23	86
	9 (34%)	4	46	72
DEAD	19 (67%)	17	27	188
	9 (56%)	9	96	180
Pkinase_Tyr	19 (38%)			
	Not seen in ASD	17	50	301
Collagen	19 (37%)			
	Not seen in ASD	5	28	59
Tubulin	18 (100%)	13	46	227
	2 (50%)	5	113	227
I-set	18 (37%)			
	Not seen in ASD	10	22	99
Helicase_C	18 (35%)	7	41	91
	3 (12%)	3	55	76
Mito_carr	17 (73%)	14	26	146
	13 (65%)	10	50	136
UQ_con	7 (87%)	8	28	144
	12 (100%)	11	69	157

^$^, presents the number of genes that encode the domain as undergoing truncation event among the protein isoforms. In brackets, is given in terms of percentage fraction of genes that encode the domain in the protein isoforms. Values with Vega data set is shown in line 1, and values with ASD data set is shown in line 2.

^@^, the observed lengths were grouped in a manner that any two lengths that differ by 5 or less amino acids is considered as one unique length.

#### Insertion/Deletion of domains

We find that 933 of 2057 distinct annotated domains in Vega (ASD: 673 of 1592 distinct annotated domains) undergo insertion/deletion. Table 3 lists the top 20 domains that are often inserted or deleted among protein isoforms. Examination of Gene Ontology (GO) terms [41] and Pfam descriptions associated with these domains reveals that the top three affected molecular processes are: (i) regulation of transcription, as indicated by the appearance of nucleic acid binding domains (such as zf-C2H2, KRAB, WD40, RRM_1, and Helicase_C). (ii) signal transduction as indicated by the appearance of domains such as WD40, PDZ, PH, C2, CH, and SH3_1; and (iii) protein-protein interaction as indicated by the appearance of domains such as Ank, LRR_1, LIM, and KRAB. Apart from these three major categories, we find cellular adhesion & recognition (as indicated by the appearance of the Sushi, ig, collagen, C1-set, EGF, I-set, and domains), and proteolysis as affected by domain insertion/deletion events. These functional "categories" (*nucleic acid binding, signal transduction and protein-protein interaction*) represent key functions that include control of gene expression, inter-cellular relationships or cellular signaling, and basic molecular interactions of many biological processes. Protein isoforms affected by such insertion/deletion splicing events probably act as molecular switches where a specific function has to be quickly switched off – as substantiated by literature reports that some spliced isoforms lacking an exon (or a domain in our study) can have antagonist effect (such as in the case of caspase-9 protease: the constitutive form of the protein induces apoptosis, while its shorter isoform acts as an inhibitor [42,43]).

**Table 3:** Top 20 Pfam domains that are often inserted or deleted among protein isoforms*.

**Pfam Domain**	No. of genes^$^	**Pfam Description of the domain**	**Associated GO terms**	**Keywords associated with the domain**
zf-C2H2	104 (75%)	Zinc finger, C2H2 type	Zinc ion binding	Nucleic Acid binding
	61 (48%)			
PH	59 (62%)	pleckstrin homology		Intracellular signaling/constituent of cytoskeleton
	22 (44%)			
Ank	54 (80%)	Ankyrin repeat		Protein-protein interaction
	25 (34%)			
ig	51 (82%)	Immunoglobulin family		Domains for cell surface recognition.
	Not seen in asd			
fn3	46 (77%)	Fibronectin type III domain		Multi-domain glycoproteins.
	6 (28%)			
SPRY	46 (77%)	SPIa and the Ryanodine receptor		
	3 (75%)			
Collagen	45 (88%)	Collagen triple helix repeat	Phosphate transport	Extracellular structural proteins
	8 (66%)			
zf-C3HC4	44 (61%)	Zinc finger, C3HC4 type (RING finger)	Protein binding, zinc ion binding	Key role in ubiquitination pathway.
	7 (50%)			
Pkinase	44 (29%)	Protein kinase domain	ATP binding, protein kinase activity, protein amino acid phosphorylation	
	1 (2%)			
PDZ	43 (66%)	PDZ domain	Protein binding	Signaling
	18 (42%)			
KRAB	42 (46%)	Kruppel-associated box present in proteins containg C2H2 fingers.	Nucleic acid binding, intracellular, DNA-dependent regulation of transcription	Protein-protein interactions
	34 (49%)			
C1-set	41 (37%)	Immunoglobulin C1-set domain		Cell-cell recognition, cell-surface receptors, muscle structure, immune system.
	1 (50%)			
WD40	40 (83)%	WD or beta-transducin repeats		Signal transduction, transcription regulation, cell cycle control, apoptosis.
	35 (38%)			
EGF	40 (83%)	Epidermal growth factor – like domain		Found in extracellular domain.
	5 (18%)			
SH3_1	40 (51%)	Src homology 3		Signal transduction
	4 (8%)			related to cytoskeletal organisation.
Sushi	39 (97%)	Complement control protein (CCP) modules, or short consensus repeats (SCR).		Complement and adhesion
	17 (73%)			
Helicase_C	32 (62%)	Helicase conserved C-terminal domain	Nucleic acid binding	Helicase
	12 (44%)			
I-set	32 (66%)	Immunoglobulin I-set domain		Cell-cell recognition, cell-surface receptors, muscle structure, immune system
	Not present in ASD			
RRM_1	31 (72%)	RNA recognition motif	Nucleic Acid binding	RNA binding
	21 (46%)			
C2	27 (61%)	Ca2+-dependent membrane-targeting module		Signal transduction/membrane trafficking
	16 (53%)			
LIM	19 (82%)	LIM domain (Binding protein)	Zinc ion binding	Interface for protein-protein interaction
	20 (74%)			
Mito_carr	21 (91%)	Mitochondrial carrier	Transport, binding, membrane	
	19 (86%)			
CH	16 (53%)	Calponin homology domain		Cytoskeletal/signal transduction
	12 (66%)			
Hormone_receptor	9 (32%)	Ligand-binding domain of nuclear hormone receptor	Transcription factor; regulation of transcription	Hormone binding
	12 (60%)			
Trypsin	20 (41%)	Trypsin	Proteolysis	Proteolytic enzyme
	11 (30%)			

^$^, presents the number of genes in which the domain is seen as undergoing domain insertion/deletion event. In brackets, is given in terms of percentage fraction of genes containing the domain – in what percentage fraction of genes (that contain the domain), the domain undergoes insertion/deletion.

*, Line 1 gives values from Vega data set and line 2 gives values from ASD data set.

#### Domain swapping

Variations in protein isoforms due to domain swapping are less frequent as compared to domain insertion/deletion and truncation events. We identified 65 instances of protein isoform pairs (See additional file 2: Additional File 2 for the list of these protein isoform pairs) wherein a domain alternates with another. These 65 instances (3 from ASD data set and 62 from Vega data set) form a list of 35 unique pairs of alternating domains (see Table 4). Though the isoform sequences show repeats of domains in 59 of these 65 instances of isoform pairs, it is fair to believe that the domains patterns can be unambiguously aligned to extract the swap events (we have marked these instances in the database with a note as containing repeats). Examination of the description of the alternating domains (Table 4) reveals that a domain alternates often with a domain of same structural or functional classification; swapping between such similar domains probably fine-tune the biological process – some of these exemplary pairs are: (Hormone_receptor, zf-C4; KRAB, zf-C2H2; SCAN, zf-C2H2Ion_trans, Ion_trans_2; ig, I-set; I-set, V-set, EGF_CA, EGF, etc).

**Table 4:** Unique pairs of alternating Pfam domains

**Domains participating in Swap Events (D1 <--> D2)**			
Domain **D1**	Domain **D2**	Description of Domain **D1**	Description of Domain **D2**
Hormone_receptor	zf-C4	Ligand-binding domain of nuclear hormone receptor. Steroid hormone receptor activity; transcription factor activity. DNA-dependent regulation of transcription.	Zinc finger C4 type. Found in steroid/thyroid hormone receptors; transcription factor activity. Regulation of transcription.
KRAB	Zf-C2H2	Kruppel-associated box. Nucleic Acid binding; DNA dependent regulation of transcription.	Zinc finger. Nucleic acid binding.
SCAN	zf-C2H2	SCAN domain (named after SRE-ZBP, CTfin51, AW-1 and Number 18 cDNA). Found in several zf-C2H2 proteins. DNA dependent regulation of transcription.	Zinc finger, C2H2 type. Zinc ion binding; nucleic acid binding.
Mito_carr	efhand	Mitochondrial carrier. Transport	EF hand. Calcium ion binding. Signaling. Buffering/transport.
CH	Plectin	Calponin homology domain. Actin-binding family. Cytoskeletal/signal transduction	Plectin repeat. Found in Plakin proteins. Plasma and nuclear membrances.
sushi	CUB	Sushi domain (SCR repeat) Complement control protein (CCP) modules, or short consensus repeats (SCR). Complement and adhesion.	Structural motif in extracellular and plasma membrane-associated proteins.
RGS	PDZ	Regulator of G protein signaling domain.	PDZ domain. Protein binding. Signaling
C2	PDZ	Ca2+-dependent membrane-targeting module. Signal transduction/membrane trafficking	PDZ domain. Protein binding. Signaling
collagen	emi	Collagen triple helix repeat. Phosphate transport. Extracellular structural proteins	Found in extracellular proteins.
Nebulin	LIM	Nebulin repeat. Found in the thin filaments of striated vertebrate muscle. Actin-binding protein.	LIM domain (Binding protein). Zinc ion binding. Interface for protein-protein interaction
PH	Pkinase_Tyr	pleckstrin homology. Intracellular signaling/constituent of cytoskeleton. Pkinase_tyr supposed to contain PH domains.	Protein tyrosine kinase. Mediates the response to external stimuli.
Tubulin-binding	MAP2_projctn	Tau and MAP protein. Tubulin-binding repeat.	MAP domain (MHC class II analogue protein)
FHA	BRCT	Forkhead-associated domain. Phosphopeptide binding motif	BRCA1 C terminus domain. Phospho-protein binding protein.
NTP_transf_2	PAP_RNA-bind	Nucleotidyltransferase domain.	Poly(A) polymerase predicted RNA binding domain. Polynucleotide adenyltransferase activity.
Ion_trans	Ion_trans_2	Ion transport protein	Ion channel. Both are of same clan.
Orn_Arg_deC_N	Orn_DAP_Arg_deC	Pyridoxal-dependent decarboxylase, pyridoxal binding domain. Catalytic activity	Pyridoxal-dependent decarboxylase, C-terminal sheet domain. Catalytic activity.
MAM	ig	Adhesive function. Cellular component: membrance	Immunoglobulin domain
ig	I-set	Immunoglobulin	Immunoglobulin intermediate. Both are of same clan.
I-set	V-set	Immunoglobulin I-set (intermediate) domain. I-set and V-set are of same clan.	Immunoglobulin V-set (variable) domain.
EGF_CA	EGF	Calcium binding EGF domain.	EGF-like protein. Both are of same clan.
Hydrolase	E1-E2_ATPase	Haloacid dehalogenase-like hydrolase. Catalytic activity. Metabolic process	Hydrolase activity. ATP binding.
Radical_SAM	Mob_synth_C	Catalytic activity; iron-sulfur cluster binding.	Molybdenum cofactor synthesis C. iron, sulfur cluster binding
Aconitase	Aconitase_C	Aconitase hydratase. Lyase activity.	Aconitase hydratase. Hydro-lyase activity.
Filament	Filament_head	Intermediate filament protein. Structural molecule activity	Head region of intermediate filaments.
CNH	Pkinase	Citron and Citron kinase. Small GTPase regulator activity.	Protein kinase activity. ATP binding.
PSI	Sema	Plexin repeat. Membrane. Receptor activity	Semaphorins. Secreted and transmembrane proteins.
GTP_EFTU_D2	GTP_EFTU	Elongation factor	GTP binding. Elongation factor
PARP	WWE	Poly(ADP-ribose) polymerase. Catalyses covalent attachment of ADP-ribose to DNA binding proteins	Mediates protein-protein interactions in ubiquitin and ADP ribose conjugation system.
Sushi	An_peroxidase	Complement control protein (CCP) modules, or short consensus repeats (SCR). Complement and adhesion	Animal haem peroxidase. Peroxidase activity.
PH	Oxysterol_BP	pleckstrin homology. Intracellular signaling/constituent of cytoskeleton	Oxysterol binding protein. Steroid metabolic process
Ank	KH_1	Ankyrin repeat. Protein-protein interaction	K homology domain. RNA binding
GON	TSP_1	Proteinaceous extracellular matrix. Zinc ion binding. Metalloendopeptidase activitiy.	Thrombospondin type 1 domain. Cell adhesion
Thioredoxin	DnaJ	Participates in redox reactions.	Heat shock protein binding
Collagen	EMI	Collagen triple helix repeat. Phosphate transport process. Connective tissue structures.	EMI domain. Participates in multimerization
HECT	RCC1	HECT-domain (ubiquitin-transferase) Homologous to the E6-AP Carboxyl terminus. Ubiquitin-protein ligase; protein modification process.	Regulator of chromose condensation. Acts as a guanine-nucleotide dissociation simulator (GDS)

#### Reshuffling of domains

No reshuffling event involving domains was observed in our data sets.

#### Comparison among different events involving domains

Table 5 compares the gene and event distributions for different Pfam domains; the table illustrates a trend that certain domains show preference of an event over other types of events. Some of the domains that particularly undergo insertion/deletion events in a higher percent fraction of genes (containing the specific domain) as compared to truncation events are: zf-C2H2, PH, Ank, SPRY, KRAB, WD40, Sushi and EGF. Domains that particularly undergo truncation events in a higher percent fraction of genes (containing the specific domain) as compared to insertion/deletion events: Trypsin, Ras, MHC_1 and ABC_tran.

**Table 5:** Pfam domains and the undergoing events – Gene & events distribution^$^

		Percent fraction of genes that show the following events with the domain.		Percent fraction of events as per the following types with the domain	
Pfam domain	No. of Genes that encode the domain (in how many of these genes, the domain undergoes change)	insertion/deletion Gene-%	Truncation Gene-%	insertion/deletion Event-%	Truncation Event-%
Pkinase	149 (139)	29%	**77%**	27%	**72%**
	48 (4)	2%	6%	25%	75%
zf-C2H2	138 (106)	**75%**	1%	**98%**	1%
	127 (61)	48%	0%	**100%**	0%
C1-set	108 (86)	37%	43%	46%	53%
	2 (1)	50%	0%	100%	0%
PH	94 (73)	**62%**	25%	**71%**	28%
	50 (24)	44%	12%	**78%**	21%
Ank	67 (55)	**80%**	7%	**91%**	8%
	72 (25)	34%	5%	**86%**	13%
ig	62 (52)	**82%**	8%	**91%**	8%
	Not seen in ASD				
fn3	59 (49)	**77%**	30%	**71%**	28%
	21 (6)	28%	4%	85%	14%
SPRY	59 (48)	**77%**	5%	**93%**	6%
	4 (3)	75%	0%	100%	0%
Trypsin	47 (47)	42%	**68%**	38%	**61%**
	36 (29)	30%	**55%**	35%	**64%**
PDZ	65 (46)	**66%**	27%	**70%**	29%
	42 (20)	42%	11%	**78%**	21%
zf-C3HC4	72 (46)	**61%**	4%	**93%**	6%
	14 (7)	50%	0%	100%	0%
Collagen	51 (45)	**88%**	37%	**70%**	29%
	12 (8)	66%	0%	100%	0%
KRAB	90 (43)	46%	2%	**95%**	4%
	69 (34)	49%	1%	**97%**	2%
SH3_1	77 (43)	51%	9%	**85%**	14%
	45 (4)	8%	0%	100%	0%
WD40	48 (43)	**83%**	18%	**81%**	18%
	90 (35)	38%	2%	**94%**	5%
Sushi	40 (40)	**97%**	27%	**78%**	22%
	23 (17)	**73%**	4%	**94%**	5%
EGF	48 (40)	**83%**	8%	**90%**	9%
	27 (5)	18%	0%	100%	0%
Ras	41 (39)	4%	**92%**	5%	**95%**
	Not present in ASD				
Helicase_C	51 (38)	**62%**	35%	**64%**	36%
	27 (13)	44%	11%	**80%**	20%
RRM_1	43 (38)	**72%**	46%	**60%**	39%
	45 (24)	46%	20%	**70%**	30%
MHC_I	50 (35)	12%	**70%**	14%	**85%**
	5 (3)	0%	60%	0%	100%
ABC_tran	36 (33)	50%	**80%**	38%	**61%**
	18 (13)	38%	**61%**	38%	**61%**
zf-B_box	47 (33)	46%	25%	**64%**	35%
	14 (3)	21%	0%	100%	0%
C2	44 (32)	**61%**	36%	**62%**	37%
	30 (17)	53%	10%	**84%**	15%
LIM	23 (22)	**82%**	39%	**67%**	32%
	27 (20)	**74%**	14%	**83%**	16%
Mito_carr	23 (22)	**91%**	**73%**	55%	44%
	22 (19)	**86%**	59%	59%	40%

^$^, Line 1 gives the values from Vega data set and Line 2 gives the values from ASD data set.

Domains that particularly undergo insertion/deletion events in a higher fraction of genes (containing the specific domain) as compared to truncation events are: zf-C2H2, PH, Ank, SPRY, KRAB, WD40, Sushi, and EGF. Domains that undergo truncation events in higher fraction of genes (containing the specific domain) as compared to insertion/deletion events: Trypsin, Ras, MHC_1, and ABC_trans. Since Swap events were seen in few instances, they are not considered in deriving this table

### Use of both PRINTS and Pfam resources for annotating the protein isoforms

Examination of the genes and isoforms from Set D (that is used for the analysis) indicate that PRINTS could annotate 898 Vega (774 ASD) genes with detectable changes in fingerprints among isoforms, and Pfam could annotate 4583 Vega (2375 ASD) genes with detectable changes in domains among isoforms. While only in the case of 9 Vega and 27 ASD genes none of the encoded protein isoforms could be annotated for Pfam domains, in the case of 2729 Vega and 1466 ASD genes none of the encoded protein isoforms could be annotated for PRINTS fingerprints. As mentioned through in earlier sections, the observations/interpretations (*e*.*g*. truncations being the predominant event, and types of domains & molecular processes being most affected) from the analysis of Pfam or PRINTS have been supporting and complementing each other.

### Orphan protein isoforms?

Tress *et al *[15] find little evidence as to whether a majority of protein isoforms, as identified in the ENCODE pilot project [44], have a role as functional proteins; they find substantial alterations in the 3-dimensional structures of as high as 49 of the 85 protein isoforms. It has been reported that there can be large conformational changes among protein isoforms in 67% instances of alternatively spliced genes [6]. Talavera *et al *[45] find that alternative splicing affects protein sequence and structure in a more drastic way as compared with other similar events (such as gene duplication & divergence) that bring about diversity in proteins. Takeda *et al *[39] find that in 20% instances of alternatively spliced human genes, the protein products lacked annotation for protein motifs. Further, it is known that pipelines such as ASD use EST/mRNA sequences from a variety of clones/CDNA libraries that are derived from either healthy or diseased or even pooled tissues; and curated data sets contain transcript/protein isoforms that are expressed in diseased states of the cell; thus it is possible that some of the protein isoforms are indeed expressed in diseased states and hence may lack any function.

We set out to identify such the set of protein isoforms that we call as 'orphan' isoforms; this term refers to situations where one or more (but not all) of the protein isoforms from a gene lack any annotation for either Pfam domains or PRINTS fingerprints. As mentioned earlier, examination of the protein isoforms led to four sets with varying degrees of annotation for Pfam/PRINTS signatures; of these, the Sets B-D may contain potential orphan isoforms. However, we consider only the Set D for the reason that it includes only those genes for which two or more isoforms could be annotated and decipherable changes in signatures could be seen among the isoforms. Certain details on the nature of observed orphan protein isoforms are as discussed below:

#### (i) Length distributions of orphan isoforms

Set D for Vega data set contains a total of 18297 isoforms of which 2687 isoforms lack any annotation for either fingerprints or domains (the corresponding numbers for ASD data set are 11004 and 2628). We examined length distributions of protein isoforms and found that the average length of orphan isoforms is low at 128 amino acids (109 in the case of ASD data set) while the average length of annotated isoforms can be high at 449 amino acids (360 in the case of ASD) and that of human proteins in UniProt/SwissProt is 450 amino acids. The annotated isoforms peaked at around 125 amino acids; the distribution for the orphan isoforms was seen to be distinct from that of annotated isoforms, peaks earlier, and does not have the pronounced tail. The observed low value for the average length of orphan isoforms is in the order of typical lengths of single-domain proteins; domain lengths distribution usually peak at around 100 residues [46].

#### (ii) Threshold criteria used to annotate for Pfam domains and PRINTS fingerprints?

We have used a threshold for E-value as ≤ 10^-5^for accepting the annotation for Pfam domains and PRINTS fingerprints (see the section on Methods). Relaxing the requirement on E-value from 10^-5 ^to 10^-4^, to 10^-3^, and to 1 reduces the count of orphan isoforms seen in Vega data set by only 6%, 9% and 12%, respectively (in the case of ASD data set, there is virtually no reduction). Thus it is possible to say that the observation of orphan isoforms is not due to threshold criteria used to annotate for domains and fingerprints.

#### (iii) Quality of underlying splice patterns

The ASD pipeline uses transcript (EST/mRNA) sequences to decipher splice patterns. We find that splice patterns of at least 37% of orphan isoforms are supported by 2 or more transcript sequences, and up to 44% are supported by mRNA sequences; upon considering only those orphan isoforms of length > = 125 amino acid residues (the length at which the distribution of annotated protein isoforms was seen to peak), these values increase to 48% and 60%, respectively.

#### (iv) Transcripts corresponding to orphan isoforms and nonsense-mediated decay (NMD)

It is known that certain alternative splice events lead to transcripts that are targeted for nonsense-mediated decay [36-38]. Upon examination of the ASD splice patterns corresponding to the orphan isoforms for susceptibility to nonsense-mediated decay, it is seen that only in 5.5% instances of orphan isoforms, the transcripts are putative candidates for nonsense-mediated decay. This extent is much lower than the reported estimates (namely that one in five to one in three alternatively spliced transcripts are susceptible to NMD [36-38]). Instances of transcripts susceptible to NMD can be seen even with annotated protein isoforms – the corresponding values in the case of annotated protein isoforms are 7.9% – suggesting that the observed orphan isoforms are particularly not artifacts due to lack in validating transcript data for NMD. It is appropriate to recollect from literature that NMD machinery rarely down regulates the expression of a transcript completely; 10–30% of transcripts containing premature stop codons survive (NMD-escape) and may lead to production of physiologically relevant levels of truncated protein products [47,48].

#### (v) The orphan protein isoforms probably lack any known function

The transcript sequences (confirming the isoform splice patterns in the ASD pipeline) are derived from clone/cDNA libraries with the tissue state as normal or disease disorder or as pooled/mixed; *e*.*g*. upon querying the ASD database for the count of genes with transcripts seen expressed in normal versus neoplasia cDNA libraries, it is seen that (i) for 10477 genes, at least one of the expressed transcripts is from cDNA libraries with pathological state as normal; and (ii) in roughly equal number of genes at 9590, at least one of the expressed transcripts is from cDNA libraries with neoplasia as pathological state. Aberrantly expressed splice patterns are seen in diseased cells, such as cancer [49]; the number of aberrant splicing processes causing human disease is growing exponentially (see [50] for a review). Thus, it is quite possible that the orphan protein isoforms are seen probably as results of aberrant splicing in disease states of the cell and hence they lack annotation for signatures. It is important to note that the signatures seen in the constitutive protein (and in some of the encoded isoforms) are totally lost in orphan isoforms and hence the functions associated with the constitutive protein are lost in the orphan isoforms. Further, it is safe to say that Pfam and PRINTS are probably comprehensive enough to report signatures of 'known' functions. Hence we can say that the orphan isoforms lack any 'known' function.

#### (vi) Estimates for orphan protein isoforms

A wild estimate is one that is based on unannotated protein isoforms of all lengths. Vega data set: Of 18297 isoforms (from 4673 genes), 2687 isoforms (from 1385 genes) are orphans; ASD data set: of 11004 isoforms (from 2678 genes), 2628 isoforms (from 2628 genes) are orphans. Such a wild estimate is: From Vega data set: (a) one in every 3.4 genes can express an orphan protein that lacks any "known" function, and (b) One in every 6.8 alternative splice events can result in transcript isoform that encodes a protein lacking any "known" function; From ASD data set: one in every 1.02 genes and one in every 4.2 isoforms. A conservative estimate can be obtained by ignoring short isoforms of length < 125 residues – in Vega data set, of 13591 isoforms (from 4248 genes) of lengths > = 125 amino acid residues, 722 isoforms (from 477 genes) are orphans. The conservative estimate as seen in Vega data set is: one in 8.9 genes can be seen to lead to a protein isoform of no "known" function; and one in 18 protein isoforms can be such an orphan isoform; the corresponding numbers as seen in ASD data set are: one in 4.9 genes and one in 9.8 isoforms. We wish to emphasize that these estimates are subject to corrections for regulations, such as NMD, RNA silencing at transcript level and decay by cellular degradation machinery at the protein level; however, we believe that such corrections are probably taken care by the elimination of protein isoforms of shorter lengths in deriving the conserved estimate.

### Concerns & Caveats

Certain concerns, that may arise due to the methodologies & the nature of the data resources are discussed below.

#### (i) Repeats

Annotation of a fair number of isoforms comprises repeats of a single or multiple signatures. Delineating events from such annotation is difficult and can lead to ambiguous results. In such instances, we avoided delineation of events.

#### (ii) E-value thresholds

There can be instances where the E-values are close to the chosen threshold but still not good enough to accept the annotated domain/fingerprints and such instances can lead to identification of further events.

#### (iii) Underlying splice events

One may raise a concern that the events of domain deletion, swapping and reshuffling are unlikely produced by simple exon skipping or 5' and 3' splice events. Cassette exon events (and others such as alternating exon, and intron retention) can often be complex exon events – *i*.*e*. they often occur in association with extension/truncation of either one or both the flanking exons. It has been documented in ASD web pages, that 27% instances of the 18815 inferred cassette exons occur in complex form (see ). Of the reported 18815 cassette exon events, 13799 events occur only as simple cassette exons (SCE); 1418 events occur only as complex cassette exons (CCE); and 3589 occur in both the SCE and CCE forms. Cassette events involving successive multiple exons have also been reported. Intron retention events are not seen as very rare. Further, it is to be noted that an entire region of a domain does not have to be necessarily removed; deletion of crucial regions is enough to make the E-value of Pfam annotation not acceptable. An interesting aspect to consider for further studies relates to mechanistic connections between alterations (insertion/deletion, truncation, alternating, and reshuffle) of domains/fingerprints among protein isoforms to the types (exon extension/truncation, intron retention, cassette exon, alternating exon events) and extents ('simple' or 'complex' as defined in the ASD database) of splice events. We find interesting examples in our data set where alterations of protein signatures are not effected by variation in exons that code for such signatures but rather by variations in upstream exons that shift the reading frame; such an observation has been seen as prevalent in literature [42].

#### (iv) Concerns due to EST sequences in the ASD data set

The isoform splice patterns as inferred by the ASD pipeline are delineated from gene-transcript alignments; since these transcripts (cDNA/EST/mRNA) are from different sources and conditions, it leads to a concern that some of the inferred full-length transcripts are chimeric isoforms. However, this is not the case with the ASD pipeline for the following reasons: Portions of a chimeric transcript are generally from different chromosomes or from distant regions of the same chromosome. Chimeric transcripts usually pose problems when one assembles transcripts to derive gene structures or full-length transcripts. The ASD pipeline does not cluster transcripts to assemble full-length transcripts; the pipeline maps transcripts onto 'known' genes from Ensembl [51] and delineate the unique splice patterns. The methods adopted in the ASD pipeline take care that chimeric EST's are not considered – some of the relevant filter criteria (see [52] and the ASD online documents at  for more details) used are: (a) gene-transcript alignments that involve transcript sequences matching more than one gene are removed; (b) if a region of a transcript sequence matches more than one region of a gene, then the transcript sequence is removed; (c) transcripts that maps only to the flanking regions of a gene (considered is the Ensembl gene plus a region of 3000 bases flanking the gene) are ignored; matches in gene-transcript alignments of length less than a threshold are ignored; (d) transcript-gene alignments that contain only a single match on the gene are removed; and (e) gene-transcript alignments that show gap between matches on the transcript sequences are removed.

#### (v) Concerns due to derivation of protein sequence in the ASD data set

EST libraries have a 5' bias (*i*.*e*. a fraction of cDNA/EST sequences is truncated at the 5' end) and thus there can be possibilities that some of the identified splice patterns in computationally predicted data set are truncated at the 5' end. Identification of coding sequence as the longest open reading frame (ORF) from an ATG codon might provide a truncated protein isoform sequence. However, for reasons stated below, we believe that this concern has been addressed to a large extent, if not completely, by the methods of the ASD pipeline. It is not that the longest ORF from any ATG codon is considered; the context-sequence of such an ATG should score higher than a threshold value of the Kozak's ATG-context score [29]. The nucleotide sequences around the translation-initiation ATG codon is supposed to be distinctly different from those around the non-initiation ATG codons. In the ASD pipeline, known human mRNA sequences with experimentally determined translation-initiation codon were collected and used to define the threshold for the context score of initiation ATG codons. Use of this step (along with others such as match to a reference protein and requirement of a minimal length) is expected to eliminate truncated peptide sequences that start on any ATG on the splice pattern sequence.

### Use of Vega versus ASD databases for data on protein isoforms

In this work, we considered two distinct data types – one comprising manually curated protein isoforms from Vega and the other comprising protein isoforms as delineated from EST resources by the ASD computational pipeline. The estimates for orphan isoforms was seen much higher with ASD data set – a possible reason for this is that the ASD pipeline uses EST/mRNA transcript sequences, and as briefed earlier, a majority of the EST libraries are constructed from diseased tissues; and hence some of the observed protein isoforms are expressed only in diseased state of the cell and they probably lack any function. However, in general, both the data resources lead to similar results in terms of signatures that often undergo changes among protein isoforms. This observation builds a case for use of such computationally predicted databases that are, in general, are larger in size than the manually curated databases.

### SpliVaP DATABASE

#### Contents of the database

The presented work led to developing a database that holds data on protein isoforms with observed changes in signatures and domains. The main tables of the database are genes, protein isoforms, annotated domains & signatures, and the changes among the isoforms. Presented in the database are the genes and isoforms from Set D (see the section on "Varying degrees of annotation of protein isoforms for Pfam/PRINTS signatures"). The current release 1 of the database holds (i) 4673 Vega genes with 19,827 protein isoform sequences that are annotated with 727 distinct fingerprint signatures (of which 637 could be associated with at least one GO term) and 2057 distinct Pfam domain signatures (of which 1242 could be associated with at least one GO term); and (ii) 2678 ASD genes with 11,004 protein isoform sequences that are annotated with 590 distinct fingerprint signatures (of which 528 could be associated with at least one GO term) and 1592 distinct Pfam domain signatures (of which 1012 could be associated with at least one GO term).

#### Examination of the GO terms

Examining the GO terms (associated with the mapped fingerprints and Pfam domains in our data set) reveal the following as the oft-affected molecular functions: binding activity: (nucleic acid, protein, carbohydrate, lipid, cofactor, chromatin, steroid, nucleotide, nucleoside, selenium, oxygen); catalytic activity: (transferase, ligase, isomerase, oxidoreductase, deaminase, integrase, helicase, hydrolase, lyase, small protein activating enzyme); transcription regulator activity: (transcription activator, transcription repressor, transcription initiation factor, transcription factor, transcription cofactor, RNA polII transcription factor, two-component response regulator); structural molecule activity: (structural constituent of nuclear pore, vitelline membrane, ribosome, myelin sheath, extracellular matrix); transporter activity: (drug, nucleocytoplasmic); motor; and antioxidant activities.

#### Association with disease disorders

We made associations to disease disorders by using information from OMIM database. The association seen in our data sets between splice-mediated changes and disease genes (Event Type: No. Of genes) are as follows: FOR VEGA: Pfam domain truncation: 2281 disease genes; Pfam domain insertion/deletion: 1406 disease genes; Pfam domain swap: 28 disease genes; PRINTS Class_A insertion/deletion: 159 disease genes; PRINTS class_B insertion/deletion: 579 disease genes; PRINTS class_C insertion/deletion: 65 disease genes; PRINTS class_D insertion/deletion: 103 disease genes; and PRINTS swap: 4 disease genes. FOR ASD: Pfam domain truncation: 1319 disease genes; Pfam domain insertion/deletion: 806 disease genes; Pfam domain swap: 3 disease genes; PRINTS Class_A insertion/deletion: 145 disease genes; PRINTS class_B insertion/deletion: 516 disease genes; PRINTS class_C insertion/deletion: 90 disease genes; PRINTS class_D insertion/deletion: 141 disease genes; and PRINTS swap: 1 disease genes.

#### Association with structural templates

Search for associations between protein isoform sequences in our data set and data entries in Macromolecular Structure Database resulted in a set of 836 Vega genes (538 ASD genes). In each such gene, at least one protein isoform sequence can be associated with an MSD entry. In 699 of the 836 Vega genes (247 of the 538 ASD genes), more than one isoform sequence could be associated with structural data; except for few cases, the template entry from MSD was same for the multiple isoform sequences from a gene. Examination of these data indicated that such isoform sequences (with associations to MSD entries) are often results of protein shortenings (truncations) at either or both the N- and C-terminal ends. The data of such associations and indications of target structure data are useful to those who want to do homology modelling for studying structural effects of alternative splicing.

The data can be accessed via a web query interface available from our web site at . The interface allows the users to query the database through (i) gene names, GO terms, and keywords (on diseases, protein signatures & protein descriptions); (ii) associations with MSD entry and OMIM entry identifiers; (iii) types of changes (splice-mediated changes in PRINTS fingerprints and in Pfam domains); and (iv) against specific classes of PRINTS and Pfam definitions. Cross-references have been made to UniProt [28] for detailed protein information, Ensembl [51] for detailed genome annotation information, ASD for underlying transcript patterns, MSD for structural data & visualizations, and OMIM for information on genetic disorders. The interface provides an option to restrict the query to only the genes and isoforms (from curated data set) that are common between SpliVaP and Vega data sets.

**Figure 3:**
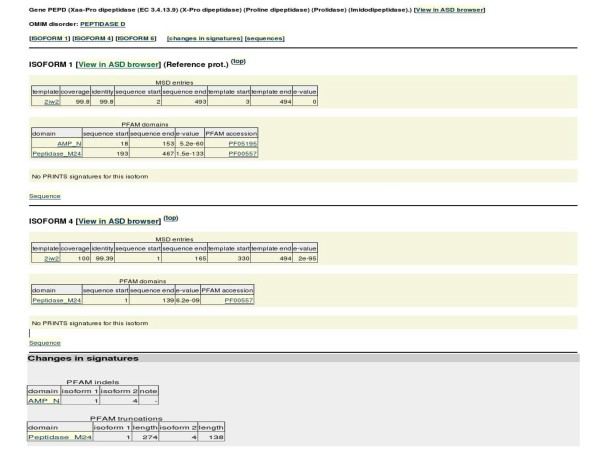
**Illustration of a typical result page from the web access of SpliVaP database.** Reported is the data on protein isoforms from PEPD gene. Reported changes in Pfam domains between two isoforms SP1 and SP4 (which are hyperlinked to splice patterns in ASD database) are an insertion/deletion and a truncation. Associations to a template structure entry in MSD, and to a related entry of genetic disorder in OMIM are shown and are hyperlinked.

Figure 3 shows an exemplary result page. Reported is the data on protein isoforms from PEPD gene. Changes (insertion/deletion and truncation of Pfam domains) are seen between two isoforms SP1 and SP4. The isoforms are hyperlinked to ASD database to show the underlying splice patterns. Association to a template structure entry in MSD, and to a related entry of genetic disorder in OMIM is shown and is hyperlinked.

### Utility of the SpliVaP Database

Several databases have been published in recent years to provide access to alternative splicing data. Some of the notable ones are HOLLYWOOD [53], ASAP II [54], H-DBAS [55], Ecgene [56], FAST-DB[57], ASTALAVISTA [58], ATD/ASD [27,59], ASPicDB [60]. Most of these databases (ASD, ASAP II, ECgene and H-DBAS) deal mainly with the collection of transcript isoforms at the nucleotide level that are then annotated with functional features such as InterPro [61] patterns, tissue specificity and literature data describing the specific isoforms. Further, databases such as Ensembl and SwissProt report splice-mediated protein variants and annotate the protein sequences for structural and functional features. Though many of these databases can be queried through features of gene and splice variants to obtain the underlying splice patterns and protein coding features, they generally do not allow the users to query for splice variants through specific changes in the composition of specific signatures (such as Pfam domains and PRINTS fingerprints) – the ability to access splice-mediated protein isoforms through changes in protein signatures (such as domain truncation or insertion/deletion) as well as the ability to obtain pre-processed information reporting changes in functional motifs among protein isoforms is missing. The SpliVaP database that we present to the community fills this gap. Thus, SpliVaP is useful for researchers in splicing community, in particular to those who are interested in studying the functional effects on protein variants. In addition, it is useful to researchers working in disease biology to access disease-associated genes that express, through alternative and aberrant splicing, proteins with altered functions – the database contains 3014 Vega genes that are associated with 2808 unique OMIM entries (ASD: 2038 genes, 2496 distinct OMIM entries). The presented association of protein isoforms with entries in structural database provides structure templates that the users can utilize for structural studies on splice-mediated changes in protein sequences. Association of protein isoform sequences with structural data entries from MSD could be made in the case of 836 Vega genes and 538 ASD genes.

## Conclusion

The work presented here considers protein variants that are (i) extracted from manually curated database of Vega, and (ii) derived by ASD computational pipeline from transcript sequences (EST/mRNA/cDNA), and reports splice-mediated changes in protein isoforms.

Protein molecular functions that are often affected by alternative splicing in our data sets are: binding activity, catalytic activity, transcription regulator activity, structural molecule activity, transporter activity, motor, and antioxidant activities; major processes that are affected are regulation of transcription, signal transduction, and protein-protein interaction. This observation gains support from previous studies (that use computationally predicted protein isoforms [6,8,62] or that use protein isoforms from curated databases [37,63]) – see [5,9] for excellent reviews). A diverse range of changes are seen among protein isoforms, from removal of a complete domain/fingerprint to truncation of a domain or removal of a component motif of a fingerprint. Signatures can be seen alternated between two protein isoforms, though at a lower frequency than other events. The presented data suggests that alternative splicing can act (i) to make proteins lose completely functionalities of specific regions or gain new/additional functionalities (through events such as insertion/deletion of fingerprints/domains), or (ii) to act as a modulator of function (through events such as truncations of domains & fingerprints, and swap between those of same classifications), or (iii) to change the protein function (through events as swap between signatures of different classifications.

The following are novel aspects: (i) Swapping of domains/signatures seems to occur often between those of same family (Structural/Functional) classifications. (ii) Pfam domains can be seen in varying lengths among protein isoforms, and fingerprints can be seen with varying number of constituent motifs among protein isoforms; since such a variation is seen in a large number of genes and protein isoforms, it could be a general mechanism to modulate the protein function among isoforms. The observation of truncation events gain support from studies by others – *e*.*g*. Kriventseva *et al *[63] find that disruption of sequence forming a domain (similar to domain truncations) is seen in considerable fraction (up to 28%) of splice variants. (iii) We speculate that some of the splice-mediated protein isoform products may lack any "known" function and such proteins isoforms are probably expressed in disease states of tissues; a conservative estimate using data from the manually curated Vega is that one in 9 genes can lead to a protein isoform of no "known" function; and one in 18 expressed protein isoforms can be such an orphan isoform; the corresponding numbers as seen with computationally predicted ASD data set are: one in 5 genes and one in 10 isoforms.

The resultant data of protein isoforms that are annotated for splice-mediated changes is presented to the community as SpliVaP database through web query interfaces. Data on protein variants are cross-referenced to underlying transcript patterns, genome context, genetic disorders, and structural data. It is our intention to update the database regularly and expand in functionalities. A particularly important expansion in functionalities is to develop an automated procedure for extracting structural information of alternatively spliced peptide regions and to include in the database.

## Availability and requirements

Release 1 of the SpliVaP data, presented in this manuscript, is available from . Enquiries on accessing the data can be mailed to splivap@crs4.it.

## Abbreviations

Vega: Vertebrate genome annotation database; SpliVaP: Splice-mediated Variants of Proteins; EST: expressed sequence tag; mRNA: messenger RNA; pre-mRNA: precursor mRNA; BLAST: Basic Local Alignment Search Tool; ASD: Alternative Splicing Database; MSD: Macromolecular Structure Database; PDB: Protein Data Bank; PRINTS: Database of protein motif fingerprints; Pfam: Database of Protein Family Domain signatures; OMIM: Online Mendelian Inheritance in Man – a database of human genes and genetic disorders; GO – Gene Ontology that provides a controlled vocabulary to describe gene and gene product attributes; UniProt: Universal Protein Resource; SwissProt: Protein sequence database; InterProScan: It is a tool that scans a given protein sequence against protein signatures; Ensembl: A system that maintains automatic annotation of genomes.

## Authors' contributions

MF carried out the fingerprint analysis, part of Pfam analysis, NMD analysis, association with OMIM & other data resources, and building the database & interfaces. MO carried out the Pfam analysis. TAT is responsible for formulating and directing the research analysis and the development of the SpliVaP pipeline & database. TAT developed the manuscript with contributions coming from MF and MO.

## Supplementary Material

Additional file 1 **PRINTS fingerprints frequently participating in insertion/deletion events.** The top 10 frequently observed fingerprints that undergo insertion/deletion event (with either the whole fingerprint or some of the constituent motifs being affected) among protein isoforms. Click here for file

Additional file 2 **Swap, and reshuffle events involving Pfam domains and PRINTS fingerprints.** The observed swap and reshuffle events (along with the patterns of the isoform pairs) involving Pfam domains are listed. Click here for file
