# Supplementary material for: Splice-mediated Variants of Proteins (SpliVaP) – data and characterization of changes in signatures among protein isoforms due to alternative splicing
Source: BMC Genomics. 2008 Oct 2;9:453. doi: 10.1186/1471-2164-9-453 (PMC2573899; doi:10.1186/1471-2164-9-453)
Supplement: Additional file 1 — PRINTS fingerprints frequently participating in insertion/deletion events. The top 10 frequently observed fingerprints that undergo insertion/deletion event (with either the whole fingerprint or some of the constituent motifs being affected) among protein isoforms. [file 1471-2164-9-453-S1.pdf]

Table of PRINTS fingerprints that frequently undergo insertion/deletion events (including both the instances of the complete fingerprint or some of the constituent motifs being affected).

Fingerprint	Description	No. of genes$ (in ASD data set)	No. of genes$ (in VEGA data set)
SH2DOMAIN	SH2 domain signature	12 (46%)	7 (15%)
STROIDFINGER	C4-type steroid receptor zinc finger signature	11 (45%)	10 (26%)
STRDHORMONER	Steroid hormone receptor signature	11 (44%)	12 (31%)
P450	P450 superfamily signature	9 (40%)	4 (16%)
NRIONCHANNEL	Neurotransmitter-gated ion channel family signature	8 (44%)	4 (30%)
GPCRSECRETIN	Secretin-like GPCR family signature	7 (43%)	6 (27%)
TYRKINASE	Tyrosine kinase catalytic domain signature	7 (25%)	6 (10%)
SDRFAMILY	Short-chain dehydrogenase/reductase (SDR) superfamily signature	6 (26%)	7 (46%)
CHAPERONIN60	60 KD chaperonin signature	5 (71%)	
VITAMINDR	Vitamin D receptor (1I nuclear receptor) signature	5 (38%)	
SH3DOMAIN	SH3 domain signature	5 (12%)	9 (17%)
ADPTRNSLCASE	Adenine nucleotide translocator 1 signature	4 (28%)	
P67PHOX	Neutrophil cytosol factor 2 signature	4 (26%)	6 (31%)
RASTRNSFRMNG	Transforming protein p21 ras signature	4 (33%)	
CHYMOTRYPSIN	Chymotrypsin serine protease family (S1) signature	4 (44%)	

$, Presented is number of genes that express protein isoforms among which the fingerprint is seen as undergoing insertion/deletion event. In brackets, is given in terms of percentage fraction of genes containing the fingerprint – in what percentage fraction of genes (that encode the fingerprint), the fingerprint undergoes insertion/deletion events. Such a fractional value is seen uniformly high for each of the fingerprints ranked by absolute numbers.
