# Supplementary material for: Splice-mediated Variants of Proteins (SpliVaP) – data and characterization of changes in signatures among protein isoforms due to alternative splicing
Source: BMC Genomics. 2008 Oct 2;9:453. doi: 10.1186/1471-2164-9-453 (PMC2573899; doi:10.1186/1471-2164-9-453)
Supplement: Additional file 2 — Swap, and reshuffle events involving Pfam domains and PRINTS fingerprints. The observed swap and reshuffle events (along with the patterns of the isoform pairs) involving Pfam domains are listed. [file 1471-2164-9-453-S2.pdf]

Table of domains participating in Swap events, and listings of the protein isoforms (annotated for Pfam domains) giving rise to Swap events.

I. Table of domains participating in Swap events in 65 pairs of protein isoform sequences.

**Domains participating in Swap Events (D1 <--> D2)**			
Domain **D1**	Domain **D2**	Description of Domain **D1**	Description of Domain **D2**
Hormone_receptor	zf-C4	Ligand-binding domain of nuclear hormone receptor. Steroid hormone receptor activity; transcription factor activity. DNA-dependent regulation of transcription.	Zinc finger C4 type. Found in steroid/thyroid hormone receptors; transcription factor activity. Regulation of transcription.
KRAB	Zf-C2H2	Kruppel-associated box. Nucleic Acid binding; DNA dependent regulation of transcription.	Zinc finger. Nucleic acid binding.
SCAN	zf-C2H2	SCAN domain (named after SRE-ZBP, CTfin51, AW-1 and Number 18 cDNA). Found in several zf-C2H2 proteins. DNA dependent regulation of transcription.	Zinc finger, C2H2 type. Zinc ion binding; nucleic acid binding.
Mito_carr	efhand	Mitochondrial carrier. Transport	EF hand. Calcium ion binding. Signaling. Buffering/transport.
CH	Plectin	Calponin homology domain. Actin-binding family. Cytoskeletal / signal transduction	Plectin repeat. Found in Plakin proteins. Plasma and nuclear membrances.
sushi	CUB	Sushi domain (SCR repeat) Complement control protein (CCP) modules, or short consensus repeats (SCR). Complement and adhesion.	Structural motif in extracellular and plasma membrane-associated proteins.
RGS	PDZ	Regulator of G protein signaling domain.	PDZ domain. Protein binding. Signaling
C2	PDZ	Ca2+-dependent membrane-targeting module. Signal transduction /	PDZ domain. Protein binding. Signaling

Domains participating in Swap Events (D1 <--> D2)			
		membrane trafficking	
collagen	emi	Collagen triple helix repeat. Phosphate transport. Extracellular structural proteins	Found in extracellular proteins.
Nebulin	LIM	Nebulin repeat. Found in the thin filaments of striated vertebrate muscle. Actin-binding protein.	LIM domain (Binding protein).Zinc ion binding. Interface for protein-protein interaction
PH	Pkinase_Tyr	pleckstrin homology. Intracellular signaling / constituent of cytoskeleton. Pkinase_tyr supposed to contain PH domains.	Protein tyrosine kinase. Mediates the response to external stimuli.
Tubulin-binding	MAP2_projctn	Tau and MAP protein. Tubulin-binding repeat.	MAP domain (MHC class II analogue protein)
FHA	BRCT	Forkhead-associated domain. Phosphopeptide binding motif	BRCA1 C terminus domain. Phospho-protein binding protein.
NTP_transf_2	PAP_RNA-bind	Nucleotidyltransferase domain.	Poly(A) polymerase predicted RNA binding domain. Polynucleotide adenyltransferase activity.
Ion_trans	Ion_trans_2	Ion transport protein	Ion channel. Both are of same clan.
Orn_Arg_deC_N	Orn_DAP_Arg_deC	Pyridoxal-dependent decarboxylase, pyridoxal binding domain. Catalytic activity	Pyridoxal-dependent decarboxylase, C-terminal sheet domain. Catalytic activity.
MAM	ig	Adhesive function. Cellular component : membrance	Immunoglobulin domain
ig	I-set	Immunoglobulin	Immunoglobulin intermediate. Both are of same clan.
I-set	V-set	Immunoglobulin I-set (intermediate) domain. I-set and V-set are of same clan.	Immunoglobulin V-set (variable) domain.
EGF_CA	EGF	Calcium binding EGF domain.	EGF-like protein. Both are of same clan.
Hydrolase	E1-E2_ATPase	Haloacid dehalogenase-like hydrolase. Catalytic activity. Metabolic process	Hydrolase activity. ATP binding.
Radical_SAM	Mob_synth_C	Catalytic activity; iron-sulfur cluster binding.	Molybdenum cofactor synthesis C. iron, sulfur cluster binding
Aconitase	Aconitase_C	Aconitase hydratase. Lyase activity.	Aconitase hydratase. Hydro-

Domains participating in Swap Events (D1 <--> D2)			
			lyase activity.
Filament	Filament_head	Intermediate filament protein. Structural molecule activity	Head region of intermediate filaments.
CNH	Pkinase	Citron and Citron kinase. Small GTPase regulator activity.	Protein kinase activity. ATP binding.
PSI	Sema	Plexin repeat. Membrane. Receptor activity	Semaphorins. Secreted and transmembrane proteins.
GTP_EFTU_D2	GTP_EFTU	Elongation factor	GTP binding. Elongation factor
PARP	WWE	Poly(ADP-ribose) polymerase. Catalyses covalent attachment of ADP-ribose to DNA binding proteins	Mediates protein-protein interactions in ubiquitin and ADP ribose conjugation system.
Sushi	An_peroxidase	Complement control protein (CCP) modules, or short consensus repeats (SCR). Complement and adhesion	Animal haem peroxidase. Peroxidase activity.
PH	Oxysterol_BP	pleckstrin homology. Intracellular signaling / constituent of cytoskeleton	Oxysterol binding protein. Steroid metabolic process
Ank	KH_1	Ankyrin repeat. Protein-protein interaction	K homology domain. RNA binding
GON	TSP_1	Proteinaceous extracellular matrix. Zinc ion binding. Metalloendopeptidase activitiy.	Thrombospondin type 1 domain. Cell adhesion
Thioredoxin	DnaJ	Participates in redox reactions.	Heat shock protein binding
Collagen	EMI	Collagen triple helix repeat. Phosphate transport process. Connective tissue structures.	EMI domain. Participates in multimerization
HECT	RCC1	HECT-domain (ubiquitin-transferase) Homologous to the E6-AP Carboxyl terminus. Ubiquitin-protein ligase; protein modification process.	Regulator of chromose condensation. Acts as a guanine-nucleotide dissociation simulator (GDS)

II. Listings of the protein isoforms (annotated for Pfam domains) giving rise to the observed swap events.

II.1. Isoform pairs from ASD data set - in all the three instances, the isoforms involve domain repeats. There is a possibility of ambiguity in the alignment due to repeats.

'ENSG00000078018_1', 'Tubulin-binding', 'Tubulin-binding', 'Tubulin-binding', 'Tubulin-binding'
'ENSG00000078018_2', 'MAP2_projctn', 'Tubulin-binding', 'Tubulin-binding'
SWAP : Tubulin-binding, MAP2_projctn
--
'ENSG00000077232_2', 'Thioredoxin', 'Thioredoxin'
'ENSG00000077232_1', 'DnaJ', 'Thioredoxin'
SWAP : Thioredoxin, DnaJ
--
'ENSG00000121904_2', 'CUB', 'Sushi', 'CUB', 'Sushi', 'CUB', 'Sushi', 'CUB', 'Sushi', 'CUB', 'Sushi', 'CUB', 'Sushi', 'CUB'
'ENSG00000121904_3', 'Sushi', 'Sushi', 'Sushi', 'Sushi', 'Sushi', Sushi', Sushi', Sushi', 'Sushi', 'Sushi', 'Sushi',
SWAP : CUB, Sushi

II.2. Isoform pairs from Vega data set

II.2.1. 6 Isoform pairs from Vega data set – the isoforms do not show repeating domains and hence the Swap events do not have any ambiguities.

'OTTHUMP00000022812', 'C2', 'RGS'
'OTTHUMP00000022813', 'C2', 'PDZ'
SWAP : RGS, PDZ
--
'OTTHUMP00000178079', 'FHA', 'Nbs1_C'
'OTTHUMP00000178080', 'BRCT', 'Nbs1_C'
SWAP : FHA, BRCT
--
'OTTHUMP00000200912', 'Collagen', 'C1q'
'OTTHUMP00000122584', 'EMI', 'C1q'
SWAP : Collagen, EMI
--
'OTTHUMP00000201249', 'PAP_central', 'NTP_transf_2'
'OTTHUMP00000201247', 'PAP_central', 'PAP_RNA-bind'
SWAP : NTP_transf_2, PAP_RNA-bind
--
'OTTHUMP00000019911', 'Ion_trans', 'BK_channel_a'
'OTTHUMP00000019914', 'Ion_trans_2', 'BK_channel_a'
SWAP : Ion_trans, Ion_trans_2
--
'OTTHUMP00000009220', 'Ion_trans_2', 'KCNQ_channel'
'OTTHUMP00000009219', 'Ion_trans', 'KCNQ_channel'
SWAP : Ion_trans_2, Ion_trans
--

II. Repeating domains.

II.2.2. 56 Isoform pairs from Vega data set – the isoforms show repeating domains and hence the Swap events may have ambiguities.

'OTTHUMP00000035904', 'Orn_Arg_deC_N', 'Orn_Arg_deC_N'
'OTTHUMP00000004286', 'Orn_Arg_deC_N', 'Orn_DAP_Arg_deC'
SWAP : Orn_Arg_deC_N, Orn_DAP_Arg_deC
--
'OTTHUMP00000065228', 'ig', 'ig'
'OTTHUMP00000199573', 'I-set', 'I-set', 'I-set', 'ig', 'I-set', 'I-set', 'V-set'
SWAP : ig, I-set
--
'OTTHUMP00000199573', 'I-set', 'I-set', 'I-set', 'ig', 'I-set', 'I-set', 'V-set'
'OTTHUMP00000065227', 'I-set', 'I-set', 'I-set', 'I-set', 'ig', 'ig'
SWAP : I-set, ig
--
'OTTHUMP00000198230', 'EGF_CA', 'EGF_CA', 'GCC2_GCC3', 'GCC2_GCC3', 'GCC2_GCC3', 'CUB'
'OTTHUMP00000198231', 'EGF_CA', 'EGF', 'EGF', 'EGF', 'EGF']
SWAP : EGF_CA, EGF
--
'OTTHUMP00000198230', 'EGF_CA', 'EGF_CA', 'GCC2_GCC3', 'GCC2_GCC3', 'GCC2_GCC3', 'CUB'
'OTTHUMP00000198232', 'EGF_CA', 'EGF', 'EGF', 'EGF'
SWAP : EGF_CA, EGF
--
'OTTHUMP00000194317', 'Hormone_recep', 'Hormone_recep'
'OTTHUMP00000194316', 'zf-C4', 'Hormone_recep'
SWAP : Hormone_recep, zf-C4
--
'OTTHUMP00000029891', 'Hydrolase', 'Hydrolase', 'Cation_ATPase_C'
'OTTHUMP00000029890', 'Cation_ATPase_N', 'E1-E2_ATPase', 'Hydrolase', 'Cation_ATPase_C'
SWAP : Hydrolase, E1-E2_ATPase
--
'OTTHUMP00000016657' : 'Plectin', 'Plectin', 'Plectin', 'Plectin'
'OTTHUMP00000016654', 'CH', 'CH', 'Plectin', 'Plectin', 'Plectin', 'Spectrin', 'Spectrin', 'Spectrin', 'Spectrin', 'Spectrin', 'Spectrin', 'Spectrin', 'Spectrin', 'Spectrin', 'Spectrin', 'Spectrin', 'Spectrin', 'Spectrin', 'GAS2'
SWAP : Plectin, CH
--
'OTTHUMP00000170536', 'PH', 'Oxysterol_BP'] (LP)
'OTTHUMP00000170538', 'Oxysterol_BP', 'Oxysterol_BP'
SWAP : PH, Oxysterol_BP
--
'OTTHUMP00000076804', 'zf-C2H2', 'zf-C2H2', 'zf-C2H2', 'zf-C2H2', 'zf-C2H2', 'zf-C2H2', 'zf-C2H2', 'zf-C2H2', 'zf-C2H2', 'zf-C2H2', 'zf-C2H2', 'zf-C2H2']
'OTTHUMP00000076806', 'KRAB', 'zf-C2H2', 'zf-C2H2'
SWAP : zf-C2H2, KRAB
--
'OTTHUMP00000078314', 'KRAB', 'zf-C2H2', 'zf-C2H2', 'zf-C2H2', 'zf-C2H2', 'zf-C2H2', 'zf-C2H2', 'zf-C2H2', 'zf-C2H2', 'zf-C2H2', 'zf-C2H2'
'OTTHUMP00000078313', 'zf-C2H2', 'zf-C2H2', 'zf-C2H2', 'zf-C2H2', 'zf-C2H2', 'zf-C2H2', 'zf-C2H2', 'zf-C2H2', 'zf-C2H2', 'zf-

C2H2', 'zf-C2H2'
SWAP : KRAB, zf-C2H2
--
'OTTHUMP00000078320', 'KRAB', 'SCAN'
'OTTHUMP00000078321', 'KRAB', 'zf-C2H2', 'zf-C2H2', 'zf-C2H2', 'zf-C2H2'
SWAP : SCAN, zf-C2H2
--
'OTTHUMP00000078321', 'KRAB', 'zf-C2H2', 'zf-C2H2', 'zf-C2H2', 'zf-C2H2'
'OTTHUMP00000078319', 'KRAB', 'SCAN'
SWAP : zf-C2H2, SCAN
--
'OTTHUMP00000195792', 'Hormone_recep', 'Hormone_recep'
'OTTHUMP00000029871', 'zf-C4', 'Hormone_recep'
SWAP : Hormone_recep, zf-C4
--
'OTTHUMP00000163915', 'MAP2_projctn', 'Tubulin-binding', 'Tubulin-binding', 'Tubulin-binding'
'OTTHUMP00000165185', 'Tubulin-binding', 'Tubulin-binding', 'Tubulin-binding', 'Tubulin-binding']
SWAP : MAP2_projctn, Tubulin-binding
--
'OTTHUMP00000174758', 'Ank'
'OTTHUMP00000159882', 'Ank', 'Ank', 'Ank', 'Ank', 'Ank', 'Ank', 'KH_1'
SWAP : Ank, KH_1
--
'OTTHUMP00000174761', 'Ank', 'Ank', 'Ank', 'Ank', 'Ank', 'Ank', 'Ank', 'Ank', 'Ank', 'Ank', 'Ank', 'Ank', 'Ank'
'OTTHUMP00000159882', 'Ank', 'Ank', 'Ank', 'Ank', 'Ank', 'Ank', 'KH_1'] (LP)
SWAP : Ank, KH_1
--
'OTTHUMP00000174762', 'Ank', 'Ank', 'Ank', 'Ank', 'Ank', 'Ank', 'Ank', 'Ank', 'Ank', 'Ank', 'Ank'
'OTTHUMP00000159882', 'Ank', 'Ank', 'Ank', 'Ank', 'Ank', 'Ank', 'KH_1'
SWAP : Ank, KH_1
--
'OTTHUMP00000016354', 'Radical_SAM', 'Mob_synth_C', 'MoaC'
'OTTHUMP00000016358', 'Mob_synth_C', 'Mob_synth_C'
SWAP : Radical_SAM, Mob_synth_C
--
'OTTHUMP00000016354', 'Radical_SAM', 'Mob_synth_C', 'MoaC'
'OTTHUMP00000016359', 'Mob_synth_C', 'Mob_synth_C'
SWAP : Radical_SAM, Mob_synth_C
--
'OTTHUMP00000194938', 'TSP_1', 'TSP_1', 'ig', 'I-set', 'I-set', 'I-set', 'TSP_1', 'PLAC'
'OTTHUMP00000194939', V-set', 'I-set'
SWAP : I-set, V-set
--
'OTTHUMP00000197355', 'RCC1', 'RCC1', 'RCC1', 'RCC1', 'HECT'
'OTTHUMP00000197360', 'RCC1', 'RCC1', 'RCC1', 'RCC1', 'RCC1', 'RCC1'
SWAP : HECT, RCC1
--
'OTTHUMP00000176016', 'Aconitase', 'Aconitase'

'OTTHUMP00000176017', 'Aconitase', 'Aconitase_C'
SWAP : Aconitase, Aconitase_C
--
'OTTHUMP00000167334', 'Filament', 'Filament'
'OTTHUMP00000167332', 'Filament_head', 'Filament'
SWAP : Filament, Filament_head
--
'OTTHUMP00000193482', 'zf-C4', 'Hormone_recep'
'OTTHUMP00000193483', 'Hormone_recep', 'Hormone_recep'
SWAP : zf-C4, Hormone_recep
--
'OTTHUMP00000019269', 'Nebulin', 'Nebulin', 'Nebulin', 'Nebulin', 'Nebulin', 'Nebulin', 'Nebulin', 'SH3_1'
'OTTHUMP00000019268', '-', '-', '-', '-', 'LIM', 'Nebulin', 'Nebulin', 'SH3_1'
SWAP : Nebulin, LIM
--
'OTTHUMP00000042793', 'Pkinase', 'CNH'
'OTTHUMP00000069728', 'Pkinase', 'Pkinase'
SWAP : CNH, Pkinase
--
'OTTHUMP00000016720', 'C2', 'C2'
'OTTHUMP00000016713', 'PDZ', 'C2'
SWAP : C2, PDZ
--
'OTTHUMP00000016718', 'C2', 'C2'
'OTTHUMP00000016713', 'PDZ', 'C2'
SWAP : C2, PDZ
--
'OTTHUMP00000016719', 'C2', 'C2'
'OTTHUMP00000016713', 'PDZ', 'C2'
SWAP : C2, PDZ
--
'OTTHUMP00000016716', 'C2', 'C2'
'OTTHUMP00000016713', 'PDZ', 'C2'
SWAP : C2, PDZ
--
'OTTHUMP00000016717', 'C2', 'C2'
'OTTHUMP00000016713', 'PDZ', 'C2'
SWAP : C2, PDZ
--
'OTTHUMP00000016715', 'C2', 'C2'
'OTTHUMP00000016713', 'PDZ', 'C2'
SWAP : C2, PDZ
--
'OTTHUMP00000078354', 'zf-C2H2', 'zf-C2H2', 'zf-C2H2', 'zf-C2H2', 'zf-C2H2', 'zf-C2H2', 'zf-C2H2', 'zf-C2H2', 'zf-C2H2', 'zf-C2H2', 'zf-C2H2'
'OTTHUMP00000078355', 'KRAB', 'zf-C2H2', 'zf-C2H2'
SWAP : zf-C2H2, KRAB
--
'OTTHUMP00000171765', 'Pep_M12B_propep', 'Reprolysin', 'TSP_1', 'ADAM_spacer1', 'GON'

'OTTHUMP00000171764', 'Reprolysin', 'TSP_1', 'ADAM_spacer1', 'TSP_1', 'TSP_1', 'TSP_1', 'TSP_1', 'TSP_1'
SWAP : GON, TSP_1
--
'OTTHUMP00000078134', 'KRAB', 'zf-C2H2', 'zf-C2H2', 'zf-C2H2', 'zf-C2H2', 'zf-C2H2', 'zf-C2H2', 'zf-C2H2', 'zf-C2H2'
'OTTHUMP00000078135', 'zf-C2H2', 'zf-C2H2', 'zf-C2H2', 'zf-C2H2', 'zf-C2H2', 'zf-C2H2', 'zf-C2H2', 'zf-C2H2', 'zf-C2H2', 'zf-C2H2', 'zf-C2H2', 'zf-C2H2', 'zf-C2H2', 'zf-C2H2', 'zf-C2H2', 'zf-C2H2', 'zf-C2H2', 'zf-C2H2', 'zf-C2H2'
SWAP : KRAB, zf-C2H2
--
'OTTHUMP00000076080','KRAB', 'zf-C2H2', 'zf-C2H2', 'zf-C2H2'
'OTTHUMP00000076081', 'zf-C2H2', 'zf-C2H2', 'zf-C2H2', 'zf-C2H2', 'zf-C2H2', 'zf-C2H2', 'zf-C2H2', 'zf-C2H2', 'zf-C2H2', 'zf-C2H2', 'zf-C2H2', 'zf-C2H2'
SWAP : KRAB, zf-C2H2
--
'OTTHUMP00000076080', 'KRAB', 'zf-C2H2', 'zf-C2H2', 'zf-C2H2'
'OTTHUMP00000076082', 'zf-C2H2', 'zf-C2H2', 'zf-C2H2', 'zf-C2H2', 'zf-C2H2', 'zf-C2H2', 'zf-C2H2', 'zf-C2H2', 'zf-C2H2', 'zf-C2H2', 'zf-C2H2', 'zf-C2H2'
SWAP : KRAB, zf-C2H2
--
'OTTHUMP00000190874', 'ig', 'I-set', 'ig', 'Pkinase_Tyr'
'OTTHUMP00000190880', 'I-set', 'I-set', 'Pkinase_Tyr'
SWAP : ig, I-set
--
'OTTHUMP00000076662', 'zf-C2H2', 'zf-C2H2', 'zf-C2H2', 'zf-C2H2', 'zf-C2H2', 'zf-C2H2', 'zf-C2H2', 'zf-C2H2', 'zf-C2H2', 'zf-C2H2'
'OTTHUMP00000076663', 'KRAB', 'zf-C2H2', 'zf-C2H2'
SWAP : zf-C2H2, KRAB
--
'OTTHUMP00000015917', 'Sema', 'PSI'
'OTTHUMP00000016569', 'Sema', 'Sema'
SWAP : PSI, Sema
--
'OTTHUMP00000200773', 'GTP_EFTU', 'GTP_EFTU_D2'
'OTTHUMP00000201789', 'GTP_EFTU', 'GTP_EFTU'
SWAP : GTP_EFTU_D2, GTP_EFTU
--
'OTTHUMP00000126953', 'C1_1', 'C1_1', 'PH', 'Pkinase'
'OTTHUMP00000201837','Pkinase_Tyr', 'Pkinase'
SWAP : PH, Pkinase_Tyr
--
'OTTHUMP00000022251', 'Mito_carr', 'Mito_carr', 'Mito_carr'
'OTTHUMP00000022252', 'efhand', 'Mito_carr'
SWAP : Mito_carr, efhand
--
'OTTHUMP00000172451', 'A1pp', 'A1pp', 'PARP'
'OTTHUMP00000172455', 'A1pp', 'A1pp', 'A1pp', 'WWE'
SWAP : PARP, WWE
--
'OTTHUMP00000172451', 'A1pp', 'A1pp', 'PARP'
'OTTHUMP00000172456', 'A1pp', 'A1pp', 'A1pp', 'WWE'

SWAP : PARP, WWE

--

'OTTHUMP00000017181', 'MAM', 'fn3', 'fn3', 'Y_phosphatase', 'Y_phosphatase'] (LP)

'OTTHUMP00000017183', 'ig', 'fn3'

SWAP : MAM, ig

--

'OTTHUMP00000115303', 'An_peroxidase', 'Sushi', 'EGF_CA'

'OTTHUMP00000115536', 'An_peroxidase', 'An_peroxidase', 'EGF_CA'

SWAP : Sushi, An_peroxidase

--

'OTTHUMP00000014912', 'zf-C4', 'Hormone_recep'

'OTTHUMP00000195566', 'Hormone_recep', 'Hormone_recep'

SWAP : zf-C4, Hormone_recep

--

'OTTHUMP00000009301', 'CH', 'CH', 'Spectrin', 'Spectrin', 'Spectrin', 'Spectrin', 'Spectrin', 'Spectrin', 'Spectrin', 'Spectrin', 'Spectrin', 'Spectrin', 'Spectrin', 'Spectrin', 'Spectrin', 'Spectrin', 'Spectrin', 'Spectrin', 'efhand', 'efhand', 'GAS2'

'OTTHUMP00000009303', 'Plectin', 'Plectin', 'Plectin', 'Plectin', 'Plectin', 'Plectin', 'Plectin', 'Plectin', '-', 'Spectrin', 'Spectrin', 'Spectrin', 'Spectrin', 'Spectrin', 'Spectrin', 'Spectrin', 'Spectrin', 'Spectrin', 'Spectrin', 'Spectrin', 'Spectrin', 'Spectrin', 'Spectrin', 'Spectrin', 'Spectrin', 'efhand', 'efhand', 'GAS2'

SWAP : CH, Plectin

--

'OTTHUMP00000078368', 'KRAB', 'zf-C2H2'

'OTTHUMP00000078367', 'zf-C2H2', 'zf-C2H2', 'zf-C2H2', 'zf-C2H2', 'zf-C2H2'

SWAP : KRAB, zf-C2H2

--

'OTTHUMP00000078369', 'KRAB', 'zf-C2H2', 'zf-C2H2', 'zf-C2H2', 'zf-C2H2', 'zf-C2H2', 'zf-C2H2'

'OTTHUMP00000078370', 'zf-C2H2', 'zf-C2H2', 'zf-C2H2', 'zf-C2H2', 'zf-C2H2', 'zf-C2H2', 'zf-C2H2', 'zf-C2H2', 'zf-C2H2', 'zf-C2H2'

SWAP : KRAB, zf-C2H2

--

'OTTHUMP00000193910', 'zf-C4', 'Hormone_recep'

'OTTHUMP00000193911', 'Hormone_recep', 'Hormone_recep'

SWAP : zf-C4, Hormone_recep

--

'OTTHUMP00000078341', 'KRAB', 'KRAB', 'zf-C2H2', 'zf-C2H2'

'OTTHUMP00000078343', 'KRAB', 'zf-C2H2', 'zf-C2H2', 'zf-C2H2', 'zf-C2H2', 'zf-C2H2', 'zf-C2H2', 'zf-C2H2', 'zf-C2H2', 'zf-C2H2', 'zf-C2H2'

SWAP : KRAB, zf-C2H2

--

'OTTHUMP00000078341', 'KRAB', 'KRAB', 'zf-C2H2', 'zf-C2H2'

'OTTHUMP00000078342', 'KRAB', 'zf-C2H2', 'zf-C2H2', 'zf-C2H2', 'zf-C2H2', 'zf-C2H2', 'zf-C2H2', 'zf-C2H2', 'zf-C2H2', 'zf-C2H2', 'zf-C2H2'

SWAP : KRAB, zf-C2H2

--

'OTTHUMP00000077559', 'zf-C2H2', 'zf-C2H2', 'zf-C2H2', 'zf-C2H2', 'zf-C2H2', 'zf-C2H2', 'zf-C2H2', 'zf-C2H2'

'OTTHUMP00000077558', 'KRAB', 'zf-C2H2', 'zf-C2H2', 'zf-C2H2'

SWAP : zf-C2H2, KRAB

III. The single Swap event observed with PRINTS fingerprints (from ASD data set)

ENSG00000157388_1 ENSG00000157388 1828 (LP) ['CACHANNEL', 'LVDCCALPHA1D', 'CACHANNEL']
ENSG00000157388_3 ENSG00000157388 208 ['LVDCCALPHA1D', 'LVDCCALPHA1']
SWAP CACHANNEL, LVDCCALPHA1

IV. The single reshuffle event observed with PRINTS fingerprints (from Vega data set)

OTTHUMP00000199126 OTTHUMG00000151097 330 (LP) ["SH2DOMAIN', 'SH3DOMAIN'] False
OTTHUMP00000199128 OTTHUMG00000151097 97 ['SH3DOMAIN', 'SH2DOMAIN'] False
RESHUFFLE ['SH2DOMAIN', 'SH3DOMAIN'] ['SH3DOMAIN', 'SH2DOMAIN']
